# Interferon Regulatory Factor 4 Recruits Immature B Cells to Signal Tertiary Lymphoid Structure Immaturity and Progression of Clear Cell Renal Cell Carcinoma

**DOI:** 10.7150/ijbs.113737

**Published:** 2025-06-09

**Authors:** Siqi Zhou, Shiqi Ye, Liang Chen, Qintao Ge, Jiahe Lu, Aihetaimujiang Anwaier, Xi Tian, Zhongyuan Wang, Shuxuan Zhu, Kun Chang, Jianfeng Yang, Tian Li, Hailiang Zhang, Dingwei Ye, Jianfeng Xiang, Wenhao Xu

**Affiliations:** 1 Department of Urology, Fudan University Shanghai Cancer Center; Department of Oncology, Shanghai Medical College, Fudan University, Shanghai, 200032, China.; 2 Shanghai Genitourinary Cancer Institute, Shanghai, 200032, China.; 3 Shanghai Jiao Tong University Affiliated Sixth People's Hospital South Campus (Shanghai Fengxian District Central Hospital), Shanghai, 201400, China.; 4 Department of Urology, Longhua Hospital, Shanghai University of Traditional Chinese Medicine, Shanghai, 200032, China.; 5 Tianjin Key Laboratory of Acute Abdomen Disease-Associated Organ Injury and ITCWM Repair, Institute of Integrative Medicine of Acute Abdominal Diseases, Tianjin Nankai Hospital, Tianjin Medical University, 8 Changjiang Avenue, Tianjin, 300100, China.; 6 Department of Interventional Oncology, Renji Hospital Affiliated to Shanghai Jiao Tong University School of Medicine, Shanghai, 200127, P.R. China; 7 Department of Urology, Ruijin Hospital, Shanghai Jiao Tong University School of Medicine, Shanghai, 200025, China.

**Keywords:** tertiary lymphoid structures (TLSs), clear cell renal cell carcinoma (ccRCC), tumor microenvironment (TME), interferon regulatory factor 4 (IRF4), single-cell RNA sequencing (scRNA-seq), spatial transcriptomics analysis

## Abstract

**Background:** Tertiary lymphoid structures (TLSs), organized clusters of immune cells within non-lymphoid tissues, significantly influence tumor progression and therapeutic response. However, their prognostic relevance and underlying regulatory mechanisms in clear cell renal cell carcinoma (ccRCC) remain insufficiently characterized.

**Methods:** We integrated transcriptomic and clinical data from 928 ccRCC patients to construct a TLS-related prognostic RiskScore using machine learning algorithms. TLS maturation heterogeneity was characterized via immunohistochemistry and multiplex immunofluorescence analyses. The functional role of interferon regulatory factor 4 (IRF4), a key regulator within the TLS gene network, was investigated using *in vitro* assays. Single-cell RNA sequencing (scRNA-seq) and spatial transcriptomics were employed to dissect the involvement of IRF4 in TLS formation and maturation.

**Results:** The derived TLS-associated signature RiskScore, comprising *CCL22, LOXL1, LIPA, ADAM8, and SAA1,* effectively stratified patients into distinct prognostic groups and showed robust associations with clinical parameters, tumor microenvironment (TME) features, and predicted immunotherapy responses. Functional assays demonstrated that IRF4 significantly enhanced the malignant phenotype of 786-O and 769-P ccRCC cells. Clinically, elevated IRF4 expression independently predicted worse patient outcomes, characterized by a predominance of immature TLS phenotypes, reduced TLS density, and diminished CD8⁺ T cell infiltration. Mechanistically, scRNA-seq analyses revealed that active IRF4 signaling was predominantly confined to immature B cell states and was inversely associated with TLS maturation trajectories. Spatial transcriptomics further confirmed IRF4 enrichment within TLS regions, notably spatially segregated from high endothelial venules (HEVs) and mature TLS compartments.

**Conclusion:** In conclusion, this study establishes a robust TLS-related prognostic signature for ccRCC and elucidates the mechanistic role of IRF4 in promoting TLS immaturity and immune dysfunction. By potentially recruiting immature B cells while impairing their maturation, IRF4 contributes to an ineffective anti-tumor immune landscape, offering a promising target for therapeutic intervention.

## Introduction

Renal cell carcinoma (RCC) represents a significant cause of cancer mortality worldwide 1-3. In 2022, it was estimated that there were over 7.37×10^4^ novel cases and 2.40×10^4^ fatalities due to RCC 4. RCC prevalence in China has increased over the past thirty years, and the rate of new diagnoses per 100,000 individuals has nearly tripled, rising from 1.16 in 1990 to 3.21 in 2019^5^. Clear cell renal cell carcinoma (ccRCC), the most frequent histological subtype, accounts for approximately 70% of all RCC cases 6. Genetic alterations in key driver genes such as VHL, SETD2, PBRM1, and BAP1 are characteristic of ccRCC, contributing to genomic instability and impaired DNA repair mechanisms, thereby potentially promoting ccRCC tumorigenesis 7.

ccRCC is characterized by significant heterogeneity, leading to variable patient prognoses and highlighting the critical need for improved biomarkers beyond traditional clinicopathological features. In recent years, considerable research effort has focused on developing molecular signatures derived from high-throughput data to better stratify patients and predict outcomes. These efforts have explored various biological signatures of ccRCC. For instance, researchers have investigated signatures linked to specific cell death pathways 8,9**,** cancer stemness and non-coding RNAs 10, tumor microenvironment (TME) and immune components 11,12. This landscape of recently proposed biomarkers underscores the diverse strategies being employed and sets the stage for introducing novel signatures, like the TLS-based one in this study, which may offer unique insights into the tumor's immune landscape and patient prognosis. Despite identifying numerous potential genomic biomarkers, the translation of these findings into routinely used tissue or blood-based molecular biomarkers for guiding clinical decision-making in ccRCC remains a significant challenge 13.

TME is an intricate and dynamic system composed of diverse cell types, including endothelial cells, fibroblasts, and immune cells 14. The TME plays a critical role in tumor initiation, progression, and metastasis, and modulates therapeutic response 15,16, being actively shaped by components such as cancer-associated fibroblasts 17. Furthermore, systemic host factors, such as the gut microbiota, and intrinsic microenvironmental conditions, such as hypoxia, are increasingly recognized as modulators of the TME 18,19. Recent research indicates that tumor-associated immunity is evident in tertiary lymphoid structures (TLSs) 20. TLSs form in nonlymphoid tissues under pathological conditions, including cancer, rather than during physiological states 21,22. Structurally, TLSs are featured by a central cluster of CD20^+^ B cells surrounded by CD3^+^ T cells, similar to the lymphoid follicles in secondary lymphoid organs (SLOs) 23. The T cell region within TLSs consists of CD4^+^ T follicular helper (Tfh) cells, CD8^+^ cytotoxic T lymphocytes, CD4^+^ T helper 1 (TH1) cells, and regulatory T cells (Tregs) 24,25. Unlike SLOs, TLSs typically lack a distinct capsule, potentially facilitating their cellular constituents to interact directly with adjacent tissue 26.

TLSs are implicated in initiating and enhancing adaptive immune responses 27,28. T cells within TLSs interact with mature dendritic cells (DCs) and B cells, inducing T cell differentiation and the development of germinal centers (GCs) 21. After B cells achieve complete maturation within TLSs, they become plasma cells that secrete high-affinity IgG and IgA, potentially increasing the tumor's response to immunotherapy 29. TLSs are increasingly recognized as crucial organizing centers for anti-tumor immunity, influencing prognosis and therapeutic response across numerous malignancies 30. Accumulating evidence across diverse malignancies, such as melanoma 31, ovarian cancer 32, non-small cell lung cancer 33, bladder cancer 34, and pancreatic cancer 35, consistently links the presence, density, and particularly the maturation state of TLSs with patient prognosis and efficacy of immune checkpoint inhibitor (ICI) therapies. Indeed, understanding the broader TME's impact is crucial, as studies highlight its pivotal role in regulating intercellular communication, shaping treatment responses, and driving resistance to chemoradiation, targeted therapy, and immunotherapy 36. However, the presence of TLSs in various tumors can differ significantly, which may lead to variable outcomes 37. Factors influencing TLS maturation, such as local metabolic constraints 38, and the specific cellular interactions within them, including T-cell activation levels 35, may contribute to this heterogeneity. In ccRCC, the different localization and maturity of TLSs can lead to different prognoses, with tumor-proximal TLSs being more mature and exhibiting better prognoses 39. Therefore, prognostic models for ccRCC incorporating TLS molecular markers hold potential clinical significance.

Interferon regulatory factor 4 (IRF4) serves as one of the genetic markers for identifying TLSs in cancers, and it fulfills a complex role in immune regulation 23. Increased IRF4 levels enhance the differentiation of CD4^+^ CD25^low^ effector T (Teff) cells and reduce T follicular helper (Tfh) cell numbers 40. Additionally, IRF4 directs effector regulatory T (Treg) cell differentiation and promotes immune suppression 41. Upregulated IRF4^+^ Treg cells within tumors are significantly linked to early tumor recurrence as well as worse disease-free survival (DFS) and overall survival (OS) 42. Although IRF4 fosters proliferation and continuous differentiation of CD8^+^ T cells 43, persistently high IRF4 can lead to CD8^+^ T cell exhaustion 44. Specifically, recent studies confirm that IRF4 upregulation following T cell activation impedes human CD8 T cell effector function, while promoting cell proliferation and PD-1 expression, contributing to an exhausted phenotype in tumor-infiltrating lymphocytes (TILs) rather than activation alone 45. Moreover, IRF4 induces M2-type macrophages 46, contributing to an immunosuppressive TME. This process can be influenced by upstream signaling 47 and by post-translational modifications 48.

Previous studies have elucidated IRF4's involvement across various tumor types. Anaplastic large cell lymphoma shows a dependency on IRF4 signaling, with MYC as a key target of IRF4^49^. Furthermore, mutations affecting IRF4-DNA binding can upregulate genes specific to human lymphomas 50. IRF4 mRNA escalates from normal tissues to oral submucous fibrosis and oral squamous cell carcinoma (OSCC), paralleling immune infiltration 51. Moreover, IRF4 acts as an oncogenic factor in human non-small cell lung cancer, partly through triggering the Notch-Akt pathway 52. In multiple myeloma (MM), IRF4 is a critical transcription factor whose activity and cellular growth effects can be deregulated by the loss of the inhibitory protein BCL7A, which normally limits its DNA binding activity 53. Due to its specific overexpression and role in mediating MM progression and survival, IRF4 is actively being pursued as a therapeutic target, with novel direct small molecule inhibitors that bind its DNA-binding domain being designed and synthesized 54 and antisense oligonucleotide-based approaches being explored to silence its expression 55. Nevertheless, the precise functions of IRF4 in ccRCC are yet to be elucidated.

This study employed an integrative approach to elucidate the complex role of TLSs in ccRCC. We developed and validated a robust TLS-related signature capable of predicting patient prognosis, TME characteristics, and immunotherapy response. Then, since IRF4 is a key TLS-associated gene with unclear functions in ccRCC, we investigated its specific contribution to tumor progression and TLS maturation. By combining *in vitro* functional assays with single-cell and spatial transcriptomics analyses, we specifically sought to determine how IRF4 expression influences B cell dynamics within the TME, contributes to TLS heterogeneity, and ultimately impacts ccRCC progression, thereby identifying potential mechanisms driving immune evasion and revealing novel therapeutic vulnerabilities.

## Materials and Methods

### Raw Data Collection and Standardization Process

This study compiled transcriptomic and clinical data for 763 ccRCC patients from several online databases, including the Cancer Genome Atlas (TCGA) database, clinical proteomic tumor analysis consortium (CPTAC) database, European Molecular Biology Laboratory (EMBL) database, and International Cancer Genome Consortium (ICGC) database, using datasets TCGA-KIRC, CPTAC-3, E-MTAB-3267, and RECA-EU. To ensure comparability and standardization, RNA sequencing data initially presented as fragments per kilobase of transcript per million mapped reads (FPKM) were converted to transcripts per million (TPM), followed by log2(TPM+1) transformation. Additionally, we mitigated the batch effect using the “ComBat” algorithm of the “sva” package. The study also incorporated genetic alteration data from the TCGA database, covering somatic mutations, copy number variations (CNVs), and tumor mutational burden (TMB). All patient consent and ethical approvals were appropriately secured in the original studies. Baseline clinical data for the patients were presented in **Table S1**.

### Consensus Clustering Analysis

Our study utilized a consensus clustering approach to divide ccRCC patients into separate subgroups according to TLS-related genes and differentially expressed genes (DEGs), respectively. We executed consensus hierarchical clustering and confirmed the ideal cluster quantity and the distribution of patients using the “ConsensusClusterPlus” package in R, performing 1000 repetitions for credibility assurance 56.

### Correlation of Clinical Traits and Immune Landscape with Molecular Profiles

Several clinical variables were taken into consideration, including patient age, gender, tumor grade, and Stage. Utilizing the “survival” and “survminer” packages in R software, we performed the Kaplan-Meier (K-M) analysis to compare prognostic outcomes 57. Then, we estimated the prevalence of immune cells within samples by implementing the CIBERSORT algorithm 58, and we pinpointed the immune cells infiltrating proportion through the single-sample gene set enrichment analysis (ssGSEA) algorithm 59. Additionally, we calculated the ESTIMATE score for each ccRCC specimen using the ESTIMATE algorithm 60, and compared immune checkpoints (ICPs) expression across subgroups.

### Functional and Pathway Enrichment Analysis

We performed Gene Ontology (GO) and Kyoto Encyclopedia of Genes and Genomes (KEGG) pathway enrichment analyses with the “clusterProfiler” package 61. Additionally, we utilized the KEGG gene set (c2.cp.kegg.v7.5.1) to execute gene set variation analysis (GSVA) to underscore functional discrepancies among clusters, based on the adjusted P value < 0.001 and the threshold of |log2-fold change (FC)| > 0.2^62^.

### Distinctive Expression and Development of the TLS-Related Predictive Signature

The “limma” package in R software was utilized to pinpoint DEGs among different TLS-gene-related clusters, setting the selection threshold at |log2-FC| > 1.25 and the adjusted P value < 0.001^63^. We then devised a prognostic scoring system (RiskScore). To recognize prognosis-related genes, univariate Cox regression analysis was performed, considering DEGs expression levels and survival data. Subsequently, we used the least absolute shrinkage and selection operator (LASSO) technique and multivariate Cox regression analysis to create an optimal predictive model. RiskScore was calculated as follows: RiskScore = h0(t) × exp (expression of CCL22 × corresponding coefficient + expression of LOXL1 × corresponding coefficient + expression of LIPA × corresponding coefficient + expression of ADAM8 × corresponding coefficient + expression of SAA1 × corresponding coefficient). Patients were then categorized into low or high RiskGroups based on the median RiskScore.

### Clinical Relevance of the Predictive Signature and Establishment of a Prognostic Outcome Prediction Nomogram

We compared clinical outcomes between the two RiskGroups by employing K-M analysis. We confirmed the predictive accuracy of the RiskScore system using a receiver operating characteristic (ROC) curve, created with the “survival ROC” package in R. Moreover, the prognostic relevance of the signature was investigated by categorizing ccRCC patients according to their clinical features.

Integrating the RiskScore with clinical parameters, we formulated a nomogram for forecasting the 1-year, 3-year, and 5-year overall survival (OS) rates of ccRCC patients, utilizing the “rms”, “regplot”, and “survival” packages in R. To determine the accuracy and robustness of the nomogram, we conducted calibration curves, time-dependent ROC curves, and decision curve analysis (DCA).

### Forecasting the Treatment Response to ICI Therapy

To forecast the response to immune checkpoint inhibitor (ICI) therapy in ccRCC patients, we utilized the tumor immune dysfunction and exclusion (TIDE) analysis. TIDE assesses two principal immune escape mechanisms: impaired T cell functionality and the inhibition of T cell infiltration with low cytotoxic T lymphocyte (CTL) counts 64. Low TIDE score patients are anticipated to have a favorable response to immunotherapy. Furthermore, we corroborated the response to immunotherapy of the TLS-related signature by analyzing the David Liu cohort, which involved 121 patients with metastatic melanoma who received anti-PD-1 blocking agent treatment (nivolumab or pembrolizumab) 65. We also validated the result with CheckMate 009 and 025 (CheckMate cohort), and 136 advanced ccRCC patients treated with nivolumab (anti-PD-1) 66.

### Specimen Collection and H&E Staining

In our study, we included a cohort of 60 patients diagnosed with ccRCC who were treated at the Department of Urology, Fudan University Shanghai Cancer Center (FUSCC, Shanghai, China). We also included clinical data for 105 ccRCC cases from the Department of Urology, FUSCC, between 2010 and 2023, to examine the relationship between IRF4 expression and prognosis. These patients underwent surgical procedures during which tissue samples were collected for further analysis. The study design and the collection and use of these tissue samples were carried out with strict adherence to the Declaration of Helsinki. The study was approved by the FUSCC Ethics Committee, and all patients provided informed permission.

To assess the extent of lymphocytic infiltration, histopathological examinations were conducted. Hematoxylin and eosin (H&E) staining was conducted on slides of both ccRCC and adjacent normal tissues following reported protocols 67, and they were carefully reviewed by two experienced pathologists independently.

### TLS Maturation Analysis with Multiplex Immunofluorescence (mIF) Staining Assays

To further investigate the tumor microenvironment 68-70, we employed independent IHC staining on adjacent slides and multiplex immunofluorescence staining assay, conducted in collaboration with Shanghai KR Pharmtech, Inc., Ltd. (Shanghai, China). Specifically, we used the 7-color multiplex immunofluorescence kit (KR Pharmtech, Inc., Ltd. (Shanghai, China) to analyze the maturation of TLSs. For the staining process, we selected a panel of specific antibodies, namely CD8 (Abcam, ab178089, 1:100), CD20 (Dako, L26, IR604), CD21 (Abcam, ab227662, 1:100), CD23 (Abcam, ab315289,1:100), CK (Abcam, ab7753, 1:100), and PD-L1 (CST, E1L3N, 13 684S, 1:400) 71,72.

The tissue slides obtained were first treated with Antibody Diluent Block buffer. Following this, the slides were incubated with the primary antibody for 40-60 minutes. Then, the slides were treated with a polymer horseradish peroxidase (HRP)-conjugated secondary antibody for 10 minutes. Next, the Fluorophore Working Solution was applied to the slides for 10 minutes, visualizing the complexes. The nuclei within the tissue slides were stained with 4',6-diamidino-2-phenylindole (DAPI). Finally, whole slide scans were conducted at 20× magnification using the KR-HT5 system (KR Pharmtech, Inc., Ltd. (Shanghai, China)). The acquired images were then analyzed using inForm 2.4.0 software.

Images were classified into TLS maturation stages as follows: early TLS (E-TLS) depicted dense lymphocytic aggregates of mixed CD8^+^T and B cells (CD8 and CD20 positive) without follicular DCs (FDCs) and GC (CD21 and CD23 signals negative); primary follicle-like TLS (PFL-TLS) showed dense lymphocytic aggregates with FDCs and absence of a GC (CD21 positive but without CD23 signals); secondary follicle-like TLS (SFL-TLS) were identified by both FDCs and a GC (CD21 and CD23 positive)^39^. TLS-positive samples in the study were categorized as follows: SFL-TLS positive, containing at least one instance of SFL-TLS; PFL-TLS positive, with at least one PFL-TLS occurrence but no SFL-TLS; E-TLS positive, exhibiting neither PFL-TLS nor SFL-TLS 39,73.

### Immunohistochemistry and Scoring Assays

After identifying at least one instance of TLSs in H&E-stained slides, adjacent slides were subjected to immunohistochemical (IHC) staining, performed as previously described 67, and IRF4 (ab315394, Abcam) was identified.

In order to quantify the intensity of IRF4 staining, an immunohistochemistry scoring system was employed, named IHCscore. The staining number score was determined based on the percentage of cells showing positive staining: a score of 0 was assigned for negative staining, 1 for 1-25% staining, 2 for 26-50% staining, 3 for 51-75% staining, and 4 for 76-100% staining. The staining color scored 0 for negative, 1 for weak, 2 for medium, and 3 for strong staining. The final IHCscore was calculated by multiplying the staining number score by the color score 74. Based on the median IHCscore, the samples were further categorized into high and low groups.

### Cell Culture and siRNA Transfection

The human ccRCC cell lines 786-O and 769-P were acquired from the Type Culture Collection Cell Bank, Chinese Academy of Sciences. These cells were nurtured in RPMI 1640 medium, supplemented with 10% fetal bovine serum and 1% penicillin/streptomycin. Cells were maintained in a 37°C incubator with 5% CO_2_. Cells were seeded in 10-cm dishes at 50% confluency one day before transfection. Transfection commenced when cell density approached 70%. Hieff Trans^®^ Liposomal Transfection Reagent (Yeasen, Shanghai, China) was used to prepare complexes with the negative control and IRF4 siRNA, according to the protocol from the manufacturer. After a 15-minute room temperature incubation, these complexes were introduced to the cells. We harvested the cells at 72 hours post-transfection for later analysis.

### Western Blotting Assay

Cells were lysed using Cell lysis buffer for Western and IP (P0013, Beyotime) supplemented with protease inhibitors. Protein concentrations were determined using the BCA assay (P0009, Beyotime). Equal amounts of protein were separated by 10% SDS-PAGE and transferred onto PVDF membranes. Membranes were blocked with 5% non-fat milk in TBST for 1 hour at room temperature. Membranes were then incubated overnight at 4°C with primary antibodies diluted in blocking buffer. The following primary antibodies were used: IRF4 (11247-2-AP, Proteintech), CXCL13 (10927-1-AP, Proteintech), BCL6 (21187-1-AP, Proteintech), PD-L1 (28076-1-AP, Proteintech), and GAPDH (60004-1-Ig, Proteintech). After washing with TBST, membranes were incubated with horseradish peroxidase (HRP)-conjugated secondary antibodies for 1 hour at room temperature. The following secondary antibodies were used: Goat anti-Rabbit IgG (H+L) (SA00001-2, Proteintech) and Goat anti-Mouse IgG (H+L) (SA00001-1, Proteintech). Bands were visualized using an enhanced chemiluminescence (ECL) detection kit (36222ES, Yeason) and imaged using ChemiDoc Imaging System (Bio-Rad).

### Cellular Functional Experiments *In Vitro*

The proliferation capability of cell lines was detected by colony formation assay and CCK-8 assay, respectively. For colony formation assay, we seeded 786-O or 769-P into 6-well plates with 1000 cells per well. After 14 days of culture in complete medium, the plates were gently washed twice with PBS, fixed with 4% paraformaldehyde for 20 minutes at room temperature, and then stained with 0.1% Crystal Violet solution for 20 minutes at room temperature. Following staining, plates were carefully washed with water to remove excess stain and allowed to air dry before being photographed on a luminous board. The colonies were quantified if they contained more than 50 cells.

CCK-8 assay was conducted in 96-well plates, with each well containing 2000 cells in 100 µl of complete culture medium. Measurements were taken on day 1, 2, 3, 4, and 5 after incubation, respectively, by CCK-8 assay kit (Beyotime). After adding 10 µl CCK-8 reagent to each well, cell culture proceeded for an additional 2 hours shielded from light. Subsequently, absorbance was measured at the wavelength of 450 nm using Microplate Spectrophotometer (BioTek Instruments Inc.).

Transwell assay was performed to assess cell invasion. Transwell chambers (Corning) coated with Matrigel (Corning) were loaded with 200 µl FBS-free culture medium containing 20,000 cells. Then, they were placed in 24-well plates, which contained 800 µl complete culture medium per well. After culturing for 24 hours, non-invaded cells remaining on the upper surface of the Transwell membrane were carefully removed using a cotton swab. The chambers were then washed with PBS, and the invaded cells on the lower surface were fixed with 4% paraformaldehyde for 20 minutes at room temperature. After fixation, the membranes were stained with 0.1% Crystal Violet solution for 20 minutes. Excess stain was removed by washing with PBS, and the membranes were allowed to air dry before being carefully excised, mounted on slides, and photographed.

Wound healing assay was used to evaluate cell migration. Cells were seeded into 6-well plates and allowed to grow until confluent, and a scratch was then made to create a wound. After 24 hours of incubation in FBS-free culture medium, the wound gap area was photographed and measured.

### Flow Cytometry Assay

Flow cytometry was performed to examine the apoptosis rate of cells. Cells were labeled with Annexin V-PE and 7-AAD (Multi Sciences) following the manufacturer's instructions. Then, detection was carried out using the LSRFortessa flow cytometer (BD Biosciences), and results were analyzed with CytExpert software.

### Single-Cell RNA Sequencing and Data Preprocessing

In this study, we collected scRNA-seq data from 19 ccRCC samples sourced from GSE207493. The single-cell data from the 10x platform were preprocessed following the standard protocol outlined in the R package Seurat 75. Doublets were eliminated using DoubletFinder^76^, and low-quality cells were filtered out based on specific criteria: cells with fewer than 200 or more than 6000 detected genes, or those with a mitochondrial gene ratio exceeding 20%. Additionally, low-expression genes, defined as those expressed in fewer than 5 cells, were also excluded. Cell cycle status was assessed using the CellCycleScoring() function, and potential interference from cell cycle effects was mitigated through the regression algorithm implemented in ScaleData(). Following data normalization and standardization, Harmony was employed to correct for batch effects, and the top 2000 highly variable genes were selected. Dimensionality reduction was executed using Principal Component Analysis (PCA), with the first 15 principal components chosen for t-SNE visualization. Cell clustering was performed using the FindClusters() function, with a resolution parameter set to 0.5. Finally, cell subpopulations were manually annotated based on classical marker genes and references from previous literature 77.

### Pseudo-time Trajectory Analysis

We extracted all B cells for further analysis and employed the R package monocle2^78^ for pseudo-time trajectory inference to elucidate potential cell state transitions. Highly variable genes were selected using the dispersionTable() function to construct the feature space necessary for trajectory building. Subsequently, the DDRTree algorithm was utilized for dimensionality reduction, and the developmental differentiation paths of cell states were computed through the reduceDimension() function. Trajectory visualization was achieved using plot_cell_trajectory(). Additionally, the dynamic expression changes of representative genes associated with B cell functional states were illustrated along the pseudo-time axis.

### Spatial Multi-omics Data Analysis

Spatial multi-omics data analysis was conducted using spatial transcriptomic data obtained from the Gene Expression Omnibus (GEO, https://www.ncbi.nlm.nih.gov/geo). A total of seven samples diagnosed with clear cell renal cell carcinoma (ccRCC) from dataset GSE175540 were included in the analysis. The R package Seurat was utilized for the management and visualization of the spatial transcriptomic (ST) data. To normalize the ST data, the SCTransform method was employed. The integration of ST data was achieved using the functions SelectIntegrationFeatures, PrepSCTIntegration, FindIntegrationAnchors, and IntegrateData. TLS scoring was performed based on TLS-related markers, and the expression of B cell-related markers was compared between TLS regions and non-TLS regions.

To evaluate the spatial distribution relationship and co-occurrence patterns between IRF4 expression and TLS-related structures, we conducted hotspot region identification and spatial proximity analysis. Utilizing the smoothed expression matrix derived from spatial transcriptomic data, we employed the Hotspot module 79 to compute hotspot scores for IRF4, TLS marker genes, and high endothelial venules (HEVs) marker genes. We then overlaid IRF4 expression levels with H&E staining images to delineate regions enriched in TLS. In the spatial co-occurrence analysis based on Squidpy module, we constructed a distance model based on conditional probability to calculate the likelihood of co-occurrence between IRF4 expression and HEV or TLS signals across various radius distances. The sample points were categorized into four groups: IRF4⁺/HEV⁺, IRF4⁺/HEV⁻, IRF4⁻/HEV⁺, and IRF4⁻/HEV⁻ (the same categorization applies to TLS analysis). Sampling was conducted within a distance range of 0 to 6000 μm, and co-occurrence curves were plotted to assess enrichment or depletion trends relative to random distribution as a function of distance. This method effectively reveals the spatial dependency between IRF4-positive cells and the TLS or HEV microenvironment.

### Statistical Analysis

We utilized R v4.2.3, Python v3.9, and GraphPad Prism v9.4.0 for statistical analysis and data visualization. We used the K-M estimator to plot survival curves, and significance was evaluated using the log-rank test. The Student's t-test was employed to assess disparities between two groups, while One-Way ANOVA was used for comparisons among three or more groups. All hypothesis tests employed a two-tailed approach, with statistical significance set at P-values less than 0.05.

## Results

### The Landscape of Expression Levels of the 39 TLS genes in ccRCC

**Figure 1:**
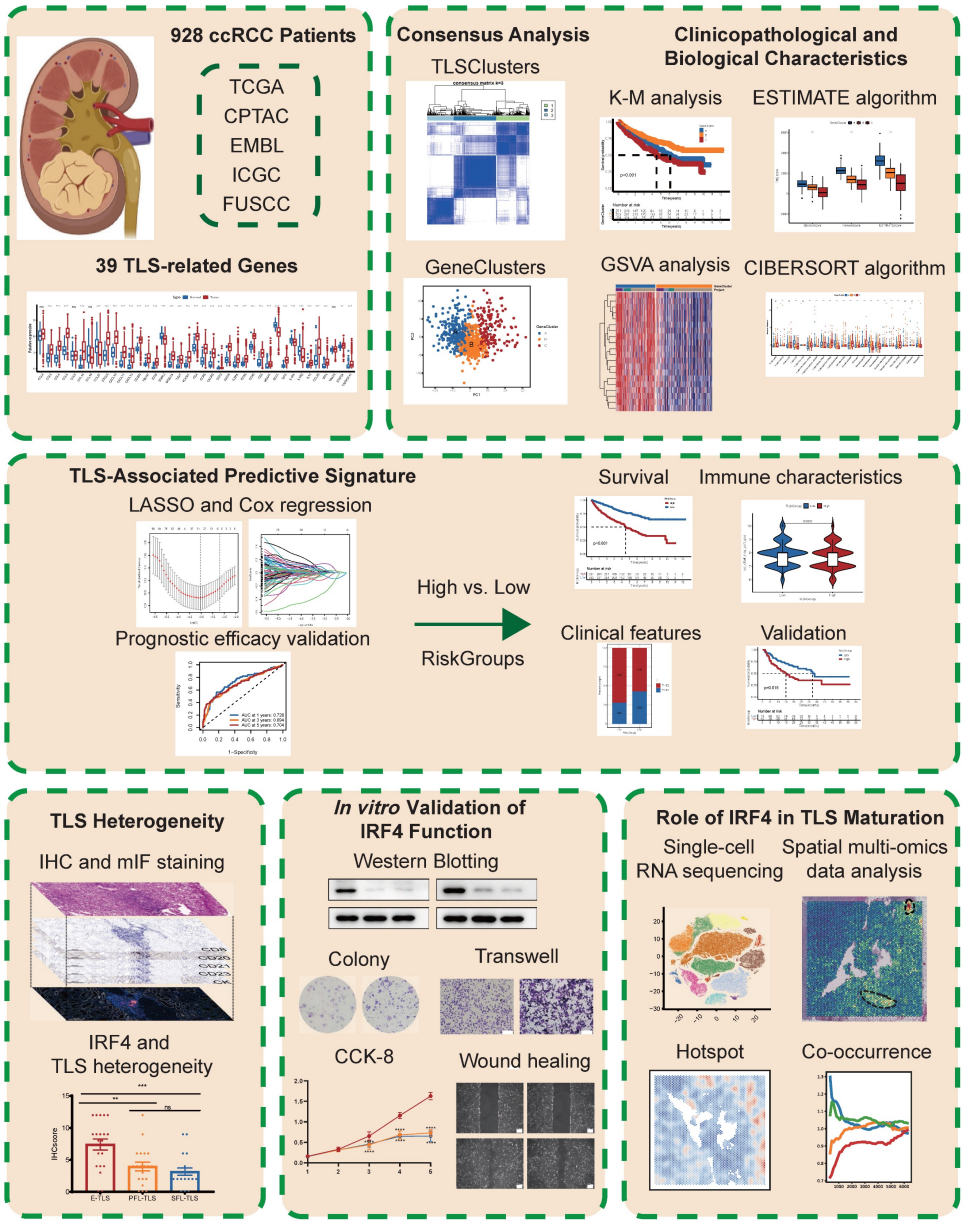
Schematic overview of the study design and main findings.

An overview of the study design and key findings is presented in **Figure 1**. We profiled 39 TLS-related genes in ccRCC, clustering patients based on expression (TLSClusters) and derived DEGs (GeneClusters), revealing distinct survival and TME profiles. Seeking to translate these insights into a more practical tool, a robust 5-gene RiskScore was developed, which independently predicted overall survival across clinical subgroups, and correlated with specific immune landscapes. To delve deeper into the biological mechanisms underlying TLS in ccRCC, we then investigated IRF4, an important TLS-related gene. We found it promotes ccRCC progression *in vitro* and correlates clinically with poor prognosis and immature TLS. Further exploring how IRF4 contributes to this phenotype, single-cell and spatial analyses revealed high IRF4 expression predominantly in immature B cells, spatially segregated from mature TLS regions and HEVs. This suggests IRF4 recruits immature B cells but hinders TLS maturation, driving immune suppression and offering a potential therapeutic target.

**Figure 2:**
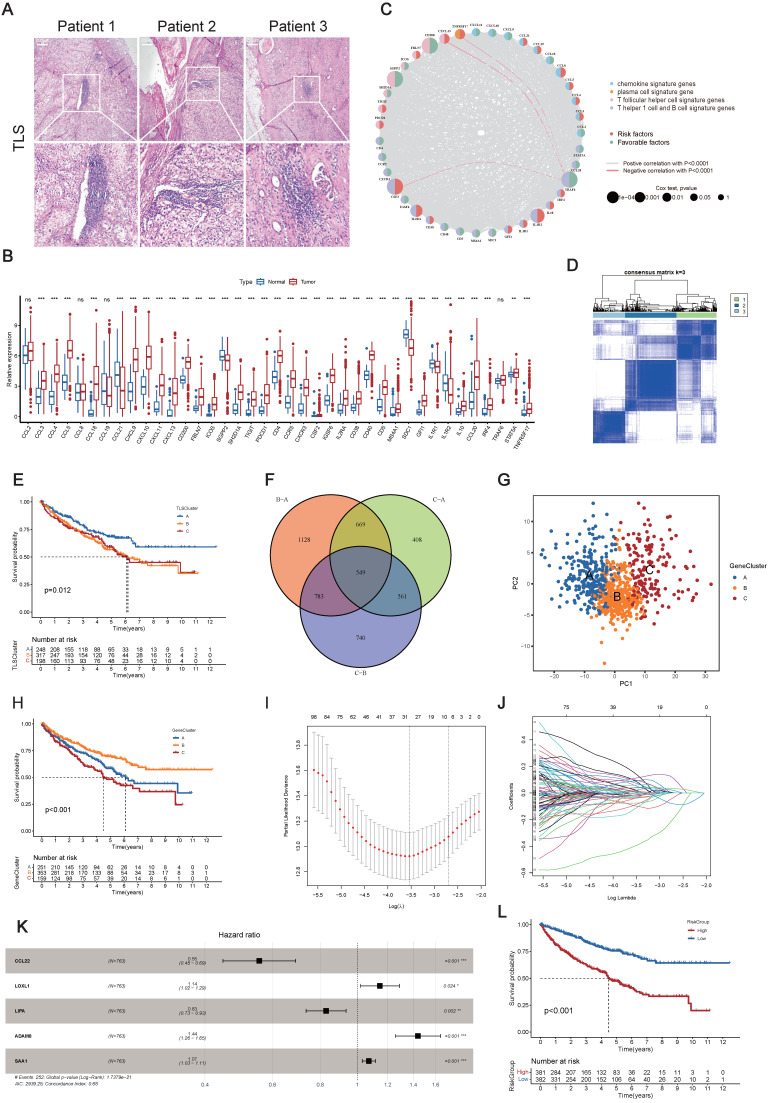
** Construction of clusters and scoring based on 39 TLS genes in ccRCC.** (A) H&E staining of tertiary lymphoid structures (TLSs) obtained from three ccRCC patients. (B) Differential expression patterns of the 39 TLS genes in tumor and normal tissues. (C) Regulatory network and prognostic significances of the 39 TLS genes. (D) Consensus matrix heatmap defining the three TLSClusters (k = 3). (E) Kaplan-Meier (K-M) curves for the overall survival (OS) of three-TLSCluster patients. (F) Venn diagram illustrating the intersection of DEGs among the three TLSClusters, with 549 DEGs common to all three. (G) PCA results showing distinct transcriptome profiling of GeneClusters. (H) K-M survival analysis showing OS prognosis across GeneClusters. (I-J) Profiles of coefficient values for 155 prognostically associated genes and identification of the optimal lambda value using the least absolute shrinkage and selection operator (LASSO) method. (K) Identification of the five genes for constructing the ideal TLS-associated prognostic model via multivariate Cox analysis. (L) K-M OS analysis for patients stratified into different RiskGroups. *, p < 0.05; **, p < 0.01; ***, p < 0.001; ns, not significant.

39-TLS related genes identified so far were analyzed in this study 23 (**Table S2**). First, we confirmed the existence of TLS in ccRCC (**Figure 2A**). Utilizing the TCGA-KIRC dataset, we evaluated the mRNA expression profiles of the 39 TLS genes. Among them, 35 TLS genes exhibited differential expression patterns in normal and tumor tissues (**Figure 2B**). The prognostic significance of the 39 TLS genes in ccRCC patients was evaluated through uniCox and Kaplan-Meier analysis (**Table S3**), demonstrating that 26 genes displayed notable disparities (**Figure S1**). Next, CNVs of the 39 TLS genes in ccRCC were assessed. CSF2 showed especially high frequencies of amplification, while CCR5, PDCD1, SGPP2, CCL20, ICOS, TIGIT, and CD200 exhibited primarily deletion (**Figure S2A**). Somatic mutations in the 39 TLS genes were found to be relatively rare in ccRCC. Among the 336 ccRCC specimens, only 11 had genetic mutations, with a frequency of 3.27% (**Figure S2B**). The aforementioned findings suggested that there were relatively rare occurrences of CNV alterations and somatic mutations in TLS genes in ccRCC. The CNVs of the TLS genes were further mapped across the 23 chromosomes in **Figure S2C**. Furthermore, a regulatory network was constructed for the 39 TLS genes in ccRCC patients, including their interactions, regulatory relationships, and survival significance (**Figure 2C** and **Table S4**).

### Clinicopathological and biological characteristics of three subgroups defined by the 39 TLS genes in ccRCC

The consensus clustering analysis was utilized to classify ccRCC patients into distinct clusters based on TLS genes, referred to as TLSClusters. The ideal cluster number was three, ascertained by the minimal crossover in the consensus matrices (**Figure S2D-N**). Among the three clusters, TLSCluster B (317) comprised the largest population, followed by TLSCluster A (248) and TLSCluster C (198) (**Figure 2D** and **Table S5**). PCA additionally substantiated the unique dispersion among groups (**Figure S3A**).

Furthermore, the survival assessment demonstrated that patients in TLSCluster A had the most favorable OS prognosis (**Figure 2E**). The ESTIMATE algorithm was used to estimate the immune and stromal fractions for each TLSCluster (**Figure S3B** and **Table S6**). The findings revealed moderate proportions of both immune and stromal cells in TLSCluster A. Furthermore, a heatmap visualized the expression levels of TLS genes and diverse clinical characteristics across the three clusters (**Figure S3C**). Besides, the GSVA analysis was conducted, which uncovered differentially distributed cancer-associated pathways in TLSClusters A-C, including B cell and T cell receptor signaling pathways (**Figure S3D-F** and **Table S7**). To evaluate the correlation between TLS genes and the TME features of ccRCC, we examined the infiltrating immune cell types across the three clusters by employing the CIBERSORT algorithm (**Table S8**). As shown in **Figure S3G**, significant differences in enrichment were observed among various immune cell populations. Furthermore, key ICPs, including CD200, CD40, TNFRSD4, NRP1, ADORA2A, and TNFRSF14, were found to be upregulated in TLSCluster A (**Figures S3H**). Moreover, we compared the clinical characteristics of these three groups, including age, gender, grade, and stage, but found no significant difference (**Figure S3I-L**). In summary, we classified three TLSClusters through the expression profiling of TLS genes, and we performed a thorough examination of the differences in survival outcomes, physiological roles, and TME features among patients assigned to these three separate TLSClusters.

### Gene Subgroups Construction Based on DEGs

Using the “limma” package, we pinpointed 549 TLSCluster-associated DEGs (**Figure 2F** and **Table S9**). To explore the complex interactions among these DEGs, a protein-protein interaction (PPI) network focusing on the top 50 DEGs was constructed (**Figure S4A**). KEGG analysis emphasized significantly enriched pathways related to cancer, including chemokine signaling pathway, Th1 and Th2 cell differentiation, and cytokine-cytokine receptor interaction (**Figure S4B** and **Table S10**), highlighting the involvement of pathways linked to the tumor immune response. Additionally, functional enrichment analyses further outlined the stimulation and growth of immune cells, along with the impact of immune molecules (**Figure S4C** and **Table S10**). Next, the uniCox analysis was employed to identify 155 genes with significant prognostic relevance (p < 0.05) (**Table S11**). Based on these genes, we employed the consensus clustering algorithm to categorize patients into distinct genetic clusters, named GeneClusters (**Figure S4D-N**, and **Table S5**). The distinct transcriptome profiling of these GeneClusters was evident in the PCA results (**Figure 2G**). Following K-M survival analysis demonstrated that individuals in GeneCluster B had the most favorable OS prognosis, while those in GeneCluster C had the poorest OS (**Figure 2H**). Additionally, **Figure S5A** illustrated the variations in clinical features among the GeneClusters, highlighting significant differences in gene expression levels across different groups and suggesting a possible correspondence between GeneClusters and TLSClusters. Next, we evaluated the TME scores within these clusters. The results indicated the highest compositions of immune and stromal cells in GeneCluster A (**Figure S5B** and **Table S6**). Furthermore, the GSVA analysis uncovered varied activation statuses of biological pathways within these subgroups (**Figure S5C-E** and **Table S12**). Notably, GeneCluster C exhibited lower enrichment of activated immune function pathways, including T cell receptor signaling pathways, B cell receptor signaling pathways, apoptosis, NK cell-mediated cytotoxicity, and chemokine signaling pathways. Ultimately, we examined the immune characteristics of the TME, encompassing immune cell types and ICPs (**Figure S5F-G** and **Table S8**). Thereafter, we compared the clinical characteristics among GeneClusters, and we found significant differences in grade and stage, while there was no difference in age and gender (**Figure S5H-K**). Among them, the proportion of low grades(G1-2) and stages (Stage I-II) in GeneCluster B is relatively high, which is consistent with the better prognosis of GeneCluster B (**Figure 2H**). Interestingly, most immune cell types showed significant differences, and all ICPs exhibited variations among the GeneClusters. These findings suggested distinct immune activity within these three GeneClusters.

### Development of a TLS-Associated Predictive Signature

Previous results underscored the crucial role of TLSs in influencing clinical outcomes, immune signatures, and TME characteristics. However, despite offering valuable insights into the patient population, these analyses did not allow for accurate prediction of outcomes for individual patients.

Given the unique complexity and diversity of TLSs, we developed a TLS-associated signature to enhance the accuracy of prognostication. We devised a rating score named RiskScore, derived from the 155 prognosis-associated DEGs. To construct an optimal predictive model, we utilized LASSO and multivariate Cox regression analyses (**Figure 2I-K**). Among the DEGs, five genes (CCL22, LOXL1, LIPA, ADAM8, and SAA1) were identified as having a significant prognostic impact (**Table S13**). Notably, CCL22 and LIPA suggested a favorable prognosis, whereas the remaining three genes showed associations with adverse outcomes. Then, patients were stratified into high and low RiskGroups according to their RiskScore. As shown in **Figure S6A**, there was a clear correlation between higher RiskScores and increased mortality. Moreover, K-M analysis confirmed that individuals characterized by a low RiskScore exhibited improved OS, aligning with the trends observed in TLSCluster A and GeneCluster B (**Figure 2E, H, and L**). The area under the curves (AUCs) of the ROC curves at 1-, 3-, and 5-years OS were 0.726, 0.694, and 0.704, respectively, showing high sensitivity and specificity (**Figure S6B**). To further substantiate the prognostic significance of the TLS-associated signature, we conducted a multivariate Cox regression analysis, incorporating variables such as age, gender, grade, stage, and RiskScore. The analysis revealed that the 95%CI for a higher RiskScore relative to a lower one ranged from 1.121 to 1.257, with a P-value<0.0001 (**Table S14**). **Figure S6C** illustrated the allocation of patients in TLSClusters, GeneClusters, RiskGroups, and clinical outcomes. It is noteworthy that patients belonging to TLSCluster A and GeneCluster B exhibited lower RiskScores (**Figure S6D-E** and **Table S5**).

### Association between Clinical Features and the TLS-Associated Predictive Signature

To rigorously assess the clinical utility of the RiskScore, we performed K-M survival analyses of various subgroups defined by different clinical features. Stratified survival analyses revealed that the disparities in forecasted outcomes between high and low RiskGroups were particularly evident in cases with advanced T stage, TNM stage, and tumor grade (**Figure S6F-G, J-K, and N-O**). Notably, patients with a high RiskScore experienced unfavorable clinical outcomes defined by T stage, TNM stage, and tumor grade. Our findings also indicated that patients in the low RiskGroup exhibited lower clinical grading (**Figure S6H-I, L-M, and P-Q**). Collectively, these results underscored the accuracy and dependability of the TLS-associated signature in forecasting clinical outcomes.

Considering the predictive strength of the RiskScore, we integrated the RiskScore alongside essential clinical features to construct a nomogram (**Figure S7A**). The calibration plot assessed the performance of the nomogram and demonstrated a remarkable concordance regarding the prognostic nomogram-estimated OS and observed OS at 1-, 3- and, 5-years (**Figure S7B**). The AUCs of the ROC curves for 1-, 3-, and 5-year OS were high (0.847, 0.802, and 0.774, respectively), suggesting an excellent predictive ability (**Figure S7C-E**). Additionally, the decision curve analysis (DCA) validated the advantageous net benefit provided by the nomogram (**Figure S7F-H**). The nomogram exhibited robust predictive power for the prognosis of ccRCC, potentially contributing to personalized clinical management.

### Investigation into Immune Characteristics of the TLS-Associated Predictive Signature

**Figure 3:**
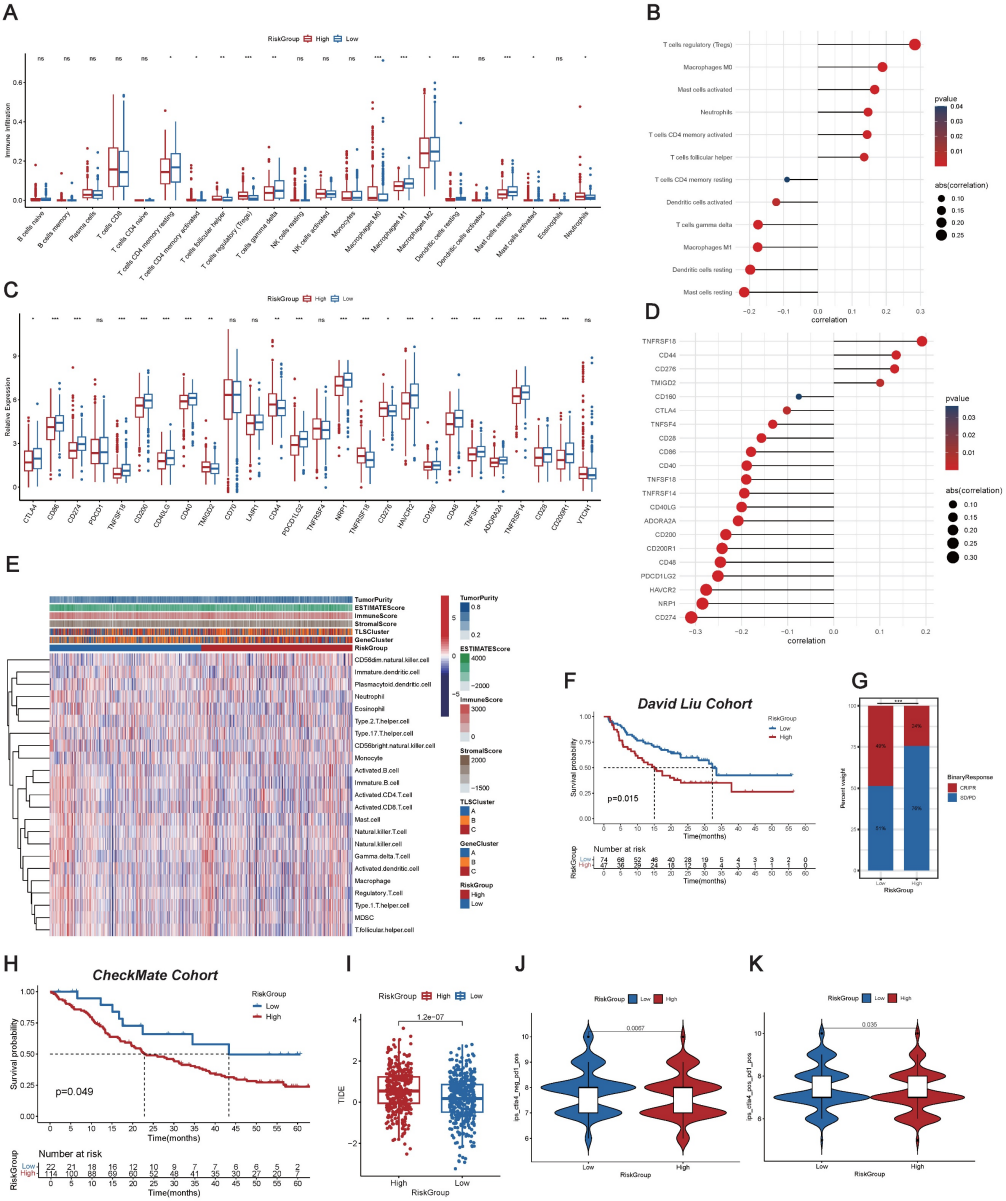
** Investigation into Immune Characteristics of the TLS-Associated Predictive Signature.** (A) Immune cell abundance across different RiskGroups. (B) Association between the RiskScore and immune cell abundance. (C) Immune checkpoint (ICP) gene expression levels within RiskGroups. (D) Correlation of the RiskScore with ICP gene expressions. (E) The heatmap illustrating the distributions of tumor microenvironment (TME) score, TLSClusters, GeneClusters, RiskGroups, and the immune cell abundance within RiskGroups. (F) K-M analyses for OS of patients with varied RiskScore in the David Liu cohort. (G) The proportion of binary response (CR/PR vs SD/PD) within distinct RiskGroups in the David Liu cohort. (H) K-M analyses for OS of patients with varied RiskScore in the CheckMate cohort. (I) Tumor immune dysfunction and exclusion (TIDE) scores within RiskGroups. (J-K) Immunophenotype Score (IPS) within different RiskGroups stratified by both CTLA4 and PD-1. *, p < 0.05; **, p < 0.01; ***, p < 0.001; ns, not significant.

We conducted a comprehensive exploration of the immune landscape among ccRCC patients within distinct RiskGroups. We initially assessed the prevalence of immune cells in ccRCC patients (**Figure 3A**). Remarkably, significant increases in infiltration within the high RiskGroup were observed, including activated T cells CD4 memory, T cells follicular helper, T cells regulatory (Tregs), macrophages M0, and neutrophils. Conversely, decreased infiltration was noted for resting T cells CD4 memory, T cells gamma delta, macrophages M1, macrophages M2, resting dendritic cells, resting mast cells, and activated mast cells. **Figure 3B** showed an association between the enrichment of immune cell types and RiskScore. Intriguingly, a significant positive correlation was detected between RiskScore and the presence of Tregs, and an inverse relationship was noted with the infiltration of resting mast cells. Considering the significance of ICI in the medical management of ccRCC patients, we examined variations in the expression profiles of ICPs across the RiskGroups (**Figure 3C**). In particular, TNFRSF18, CD44, CD276, and TMIGD2 demonstrated positive correlations with RiskScore, while other ICPs exhibited opposite correlations (**Figure 3D**). Notably, the heatmap was created to depict the patterns of tumor purity, TME scores, and the prevalence of immune-related cell varieties among RiskGroups (**Figure 3E** and **Table S15**).

Additionally, we investigated the immunotherapy outcomes among ccRCC patients. External independent confirmation using the David Liu cohort substantiated these trends, revealing a more favorable prognosis among patients in the low RiskGroup (**Figure 3F**), and the low RiskGroup exhibited a heightened proportion of CR/PR (**Figure 3G**). Similarly, we validated the above results in CheckMate Cohort, and also found that the low-risk group had a better prognosis (**Figure 3H**).

We initially determined the TIDE score for each individual, and then we observed that patients in the low RiskGroup presented with reduced TIDE scores, suggesting that patients with a low RiskScore might exhibit increased responsiveness to immunotherapy (**Figure 3I**). Furthermore, we particularly explored the importance of risk scores in assessing the impact of immunotherapy by TCIA. The findings demonstrated that the likelihood of response to CTLA4^-^/PD-L1^+^ and CTLA4^+^/PD-L1^+^ therapies was elevated in the low RiskGroup (**Figure 3J-K**). This suggested that patients in the low RiskGroup might exhibit a higher likelihood of responding to CTLA4^-^/PD-L1^+^ or CTLA4^+^/PD-L1^+^ immunotherapy, potentially leading to more satisfactory clinical outcomes. In summary, our research indicated that RiskScore was associated with various immune cells and molecules, serving as a potent predictor for anticipating ccRCC patients' response to immunotherapy.

### Insights into TLS Heterogeneity in ccRCC

**Figure 4:**
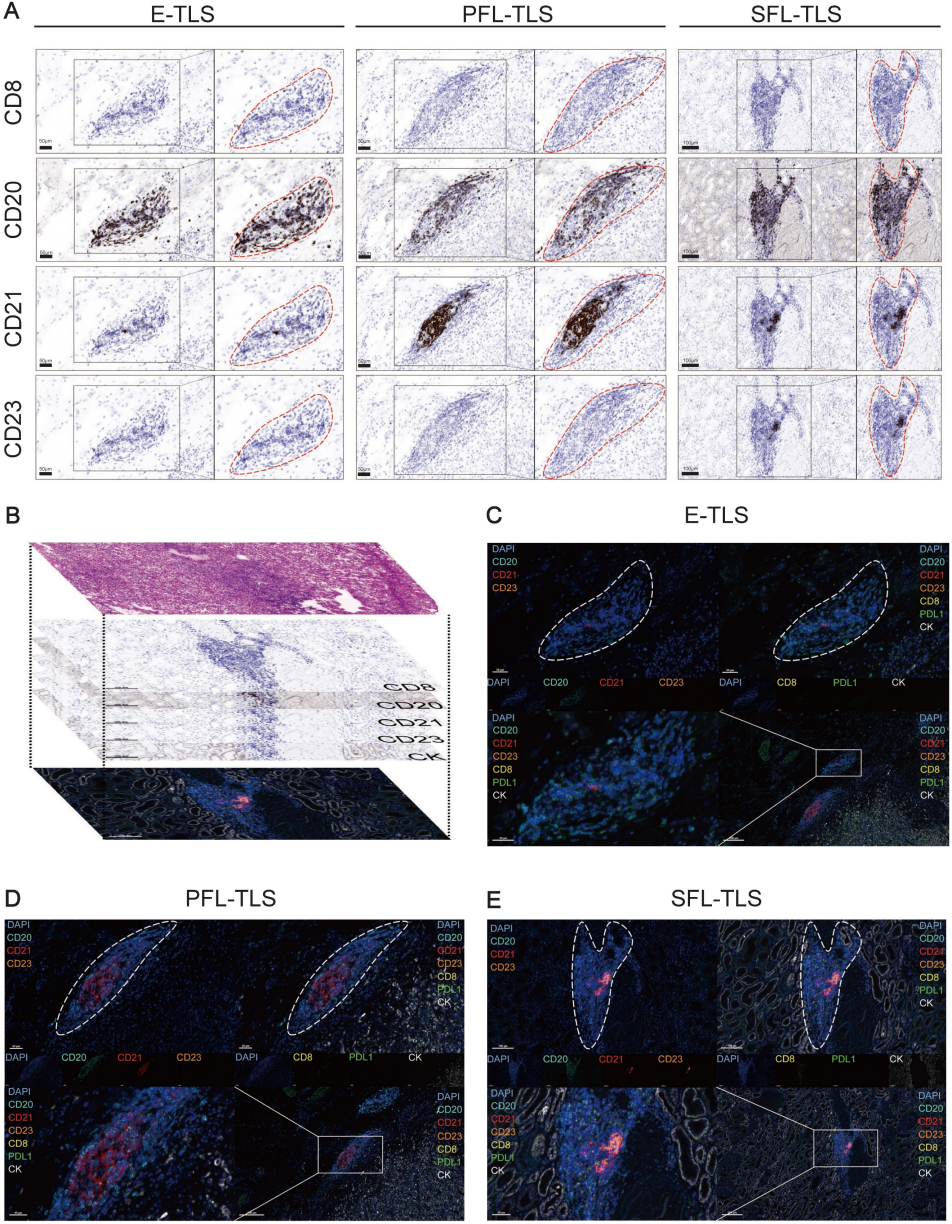
** Insights into TLS Heterogeneity in ccRCC.** (A) Continuous independent IHC results showing E-TLS, PFL-TLS, and SFL-TLS with markers CD8, CD20, CD21, and CD23. (B) Schematic diagram of H&E staining, continuous independent IHC results, and mIF in continuous slides. (C-E) mIF showing E-TLS, PFL-TLS, and SFL-TLS with markers DAPI, CD8, CD20, CD21, CD23, PD-L1, and CK.

In ccRCC, TLSs exhibited high heterogeneity 39, hence we investigated their characteristics with mIF. We preliminarily recognized the existence of E-TLS, PFL-TLS, and SFL-TLS in ccRCC by multiple independent IHC staining (**Figure 4A**). Different kinds of TLSs were identified similar to the stages of secondary lymphoid organ follicles 80. E-TLS (the first phase of TLS maturation) was defined as lymphocytic clusters without FDCs, PFL-TLS (the transitional phase of TLS maturation) as FDC-existing TLSs without GC cells, and SFL-TLS (the final phase of TLS maturation) as GC-existing clusters. Our research process was shown in **Figure 4B**: we used H&E results to initially choose regions containing TLSs, then employed IHC to ascertain their developmental stages, and finally conducted mIF for further observation. **Figure 4C-E** displayed the number and distribution of representative cells such as CD8^+^ T cells, CD20^+^ follicular B cells, CD21^+^ FDCs, and CD23^+^ GC cells in TLSs at different stages, as well as supplementing the distribution characteristics of markers such as PD-L1 and CK, at the cellular level. The above results comprehensively demonstrated the existence and characteristics of TLSs at different stages in ccRCC.

### The Oncogenic Role of IRF4 in ccRCC

IRF4 is one of the TLS-related genes 23, and it is widely regarded as a cancer-promoting gene in numerous tumors, including lung cancer, cholangiocarcinoma, and Hodgkin lymphoma 52,81,82. Nonetheless, the role of IRF4 in ccRCC is still unclear. As indicated by previous findings, IRF4 showed elevated expression in ccRCC (**Figure 2B**), and individuals exhibiting higher IRF4 expression faced a worse prognosis (**Figure S1U**). Collectively, we hypothesized that IRF4 might contribute to tumor progression in ccRCC.

**Figure 5:**
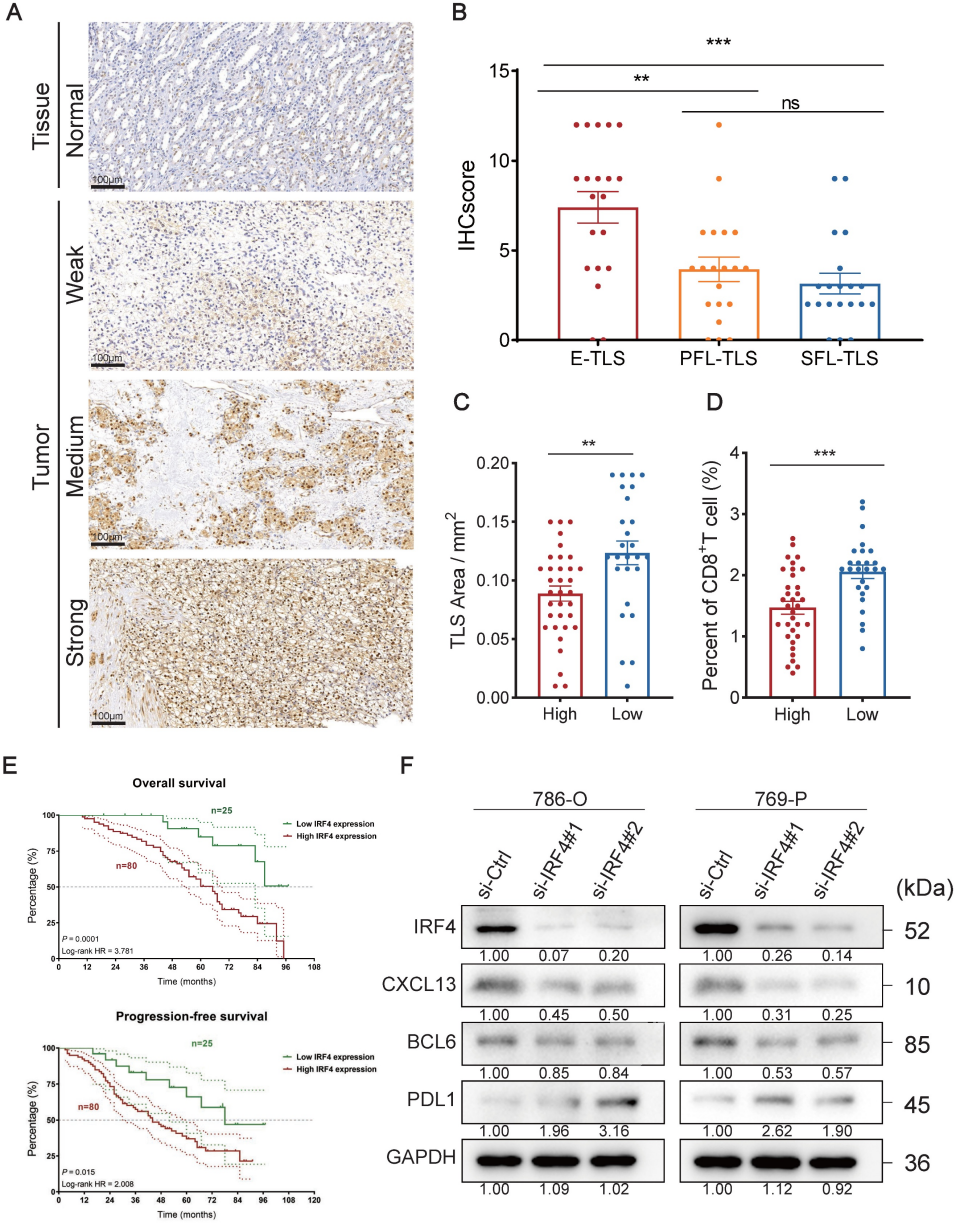
** The Oncogenic Role of IRF4 in ccRCC.** (A) IHC showing IRF4 distribution in normal tissues and tumors. (B) IHCscore in different TLS types of E-TLS, PFL-TLS, and SFL-TLS. (C-D) TLS area and percent of CD8^+^T cells in high or low IHCscore groups. (E) OS and PFS between high and low IRF4 expression group. (F) Western Blotting results of IRF4, CXCL13, BCL6, PD-L1, and GAPDH in 786-O and 769-P. *, p < 0.05; **, p < 0.01; ***, p < 0.001; ns, not significant.

We used IHC to stain the IRF4 protein of tissue and tumor slides. Typical differences in staining intensity were observed (**Figure 5A**). We calculated the corresponding IHCscore, based on the IHC staining slides, for samples containing E-TLS (n=20), PFL-TLS (n=20), and SFL-TLS (n=20) (**Figure 5B**). We observed a notable disparity in IHC score comparing E-TLS to PFL-TLS (p=0.0034), as well as a difference between E-TLS and SFL-TLS (p=0.0002), where E-TLS had the highest average IHCscore, while the difference between PFL-TLS and SFL-TLS showed no significance (p=0.3732). Next, we divided the sample into two groups, high (n=34) and low (n=26), based on the median IHCscore. We assessed the dimensions of the TLS area and the proportion of CD8^+^T cells in the specimens independently, and it was found that the elevated IHC score cohort exhibited a smaller TLS area (p=0.0039) and a diminished proportion of CD8^+^T (p=0.0003) (**Figure 5C-D**).

Furthermore, we verified whether IRF4 has a predictive effect on clinical prognosis. We included 105 ccRCC cases from FUSCC and separated them into groups of high and low levels of IRF4 RNA expression (**Figure 5E**). The results indicated that the IRF4 high expression group experienced worse OS (p=0.0001) and PFS (p=0.015) outcomes compared to the group with reduced IRF4 expression.

Thereafter, we investigated the biological function of IRF4 at a molecular level in ccRCC *in vitro*. We used human ccRCC cell lines 786-O and 769-P to construct IRF4-knockdown ccRCC cells with IRF4 siRNA. Then we detected the knockdown efficiency of IRF4 with Western Blotting, as well as the subsequent change of several TLS-related proteins such as CXCL13, BCL6, and PD-L1 (**Figure 5F**). The knockdown effect was excellent, and both CXCL13 and BCL6 showed a decreasing trend after the IRF4 knockdown, while PD-L1 increased.

**Figure 6:**
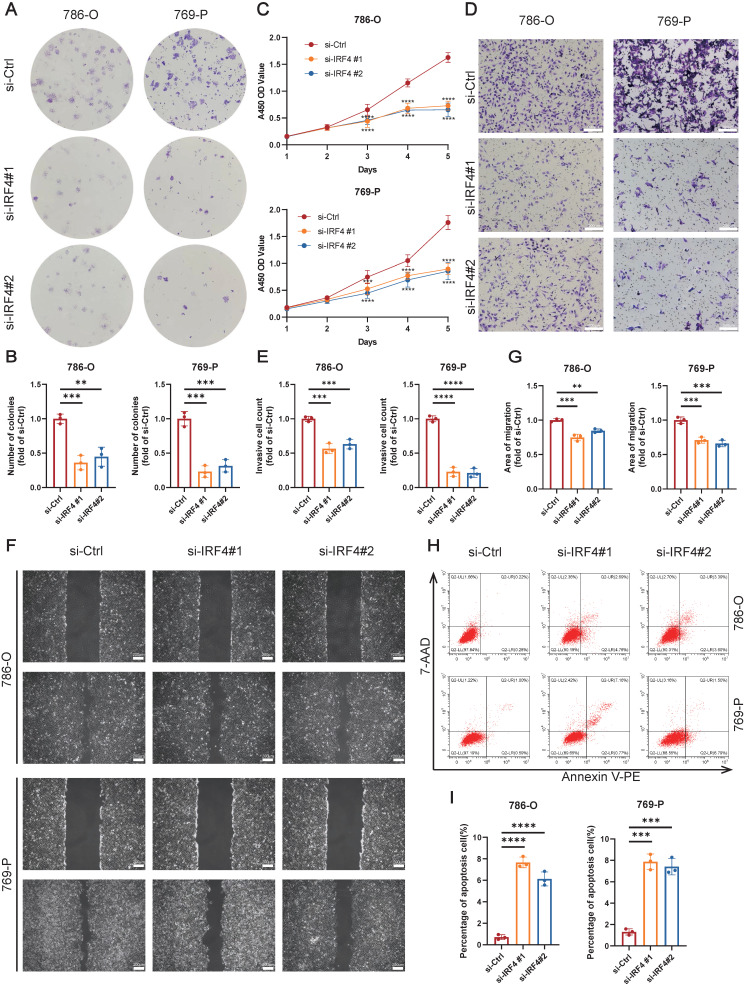
** Knockdown of IRF4 inhibiting ccRCC cell proliferation, invasion, and migration.** (A-B) The colony formation assay of 786-O and 769-P cells treated with si-Ctrl or si-IRF4. (C) The CCK-8 assay of 786-O and 769-P cells treated with si-Ctrl or si-IRF4. (D-E) The transwell assay of 786-O and 769-P cells treated with si-Ctrl or si-IRF4. (F-G) The wound healing assay of 786-O and 769-P cells treated with si-Ctrl or si-IRF4. (H-I) The apoptosis flow cytometry assay of 786-O and 769-P cells treated with si-Ctrl or si-IRF4. *, p < 0.05; **, p < 0.01; ***, p < 0.001; ns, not significant.

Finally, we explored the functional effects of IRF4 on ccRCC cells. The colony formation assay showed that knockdown of IRF4 reduced the proliferation of ccRCC (**Figure 6A-B**), and the CCK-8 assay reflected the similar results (**Figure 6C**). Deficiency of IRF4 also impaired the invasion ability of ccRCC (**Figure 6D-E**). Besides, the si-IRF4 groups showed worse migration extent in the wound healing assay (**Figure 6F-G**). Moreover, the apoptosis rate increased in IRF4-knockdown cells (**Figure 6H-I**). In summary, IRF4 may function as a cancer-promoting gene in ccRCC.

### IRF4 Marking B Cell Initiation but Restricting Maturation within the TME

The preceding findings strongly implicated IRF4 as a pro-tumorigenic driver in ccRCC. Given the observed association between high IRF4 expression and immature TLS phenotypes, we sought to explore the mechanism of this relationship. As B cells are central architects of TLSs, orchestrating their formation and maturation into germinal center-like structures, we hypothesized that IRF4 might regulate TLS development by modulating B cell differentiation or functional states within the tumor microenvironment. To explore this at the cellular level, we performed scRNA-seq on ccRCC tumor specimens.

**Figure 7:**
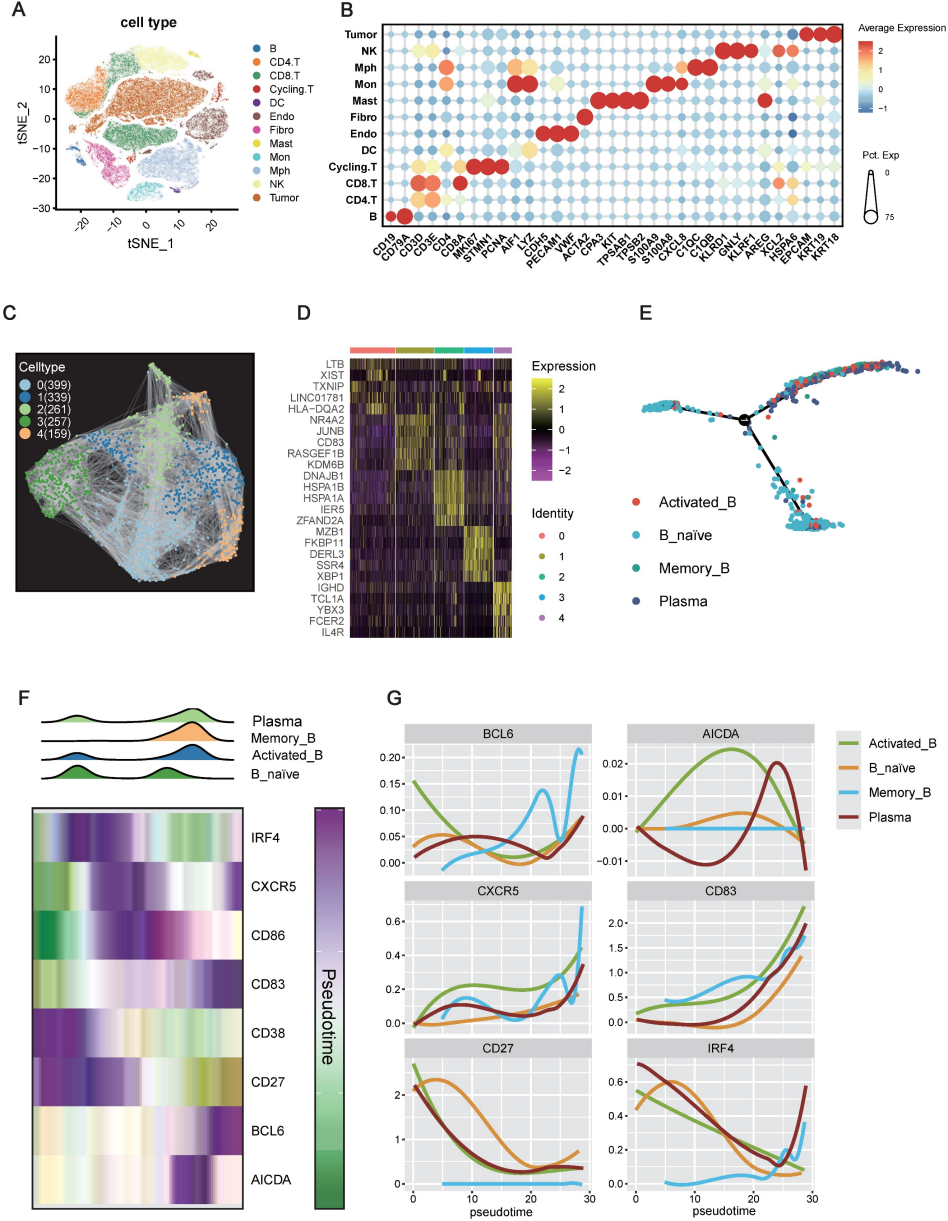
** IRF4 Marking B Cell Initiation but Restricting Maturation within the TME.** (A) t-Distributed Stochastic Neighbor Embedding (t-SNE) projection of single-cell transcriptomes from the TME. (B) Dot plot illustrating the expression patterns of selected canonical marker genes across the identified tumor and immune cell types. (C) Visualization of B cell subpopulations identified through subclustering of the total B cell. (D) Heatmap showing the top 5 differentially expressed genes across the five B cell clusters. (E) Pseudotime trajectory analysis of B cells. (F) Dynamics of B cell states and gene expression along the pseudotime trajectory. (G) Smoothed expression profiles of key B cell regulatory genes (BCL6, AICDA, CXCR5, CD83, CD27, IRF4) along the pseudotime axis.

Unsupervised clustering and t-distributed stochastic neighbor embedding (t-SNE) analyses, based on canonical immune marker genes, identified 11 immune cell populations alongside tumor cells (**Figure 7A-B**). Focusing specifically on the B cell compartment (CD19⁺, CD79A⁺), we performed re-clustering, which revealed five distinct B cell subpopulations (Clusters 0-4) (**Figure 7C-D**). Marker gene analysis allowed annotation of these clusters into four major functional subsets: Cluster 0: HLA-DQA2⁺, LTB⁺, suggestive of Memory B cells; Cluster 1: NR4A2⁺, JUNB⁺, representing Activated B cells; Cluster 2: CD83⁺, KDM6B⁺, another Memory B-enriched subset; Cluster 3: MZB1⁺, XBP1⁺, characteristic of Plasma cells; Cluster 4: IGHD⁺, FCER2⁺, indicative of Naive B cells.

To elucidate the developmental relationships among these subpopulations, we performed pseudotime trajectory analysis. The inferred trajectory suggested a differentiation continuum beginning with Naive B cells, branching toward Activated and Memory B cells, and culminating in Plasma cell fates (**Figure 7E**). The inferred trajectory suggested a potential differentiation path originating from Naive B cells, branching towards Activated or Memory B cell states and culminating in Plasma cells.

We then examined the expression dynamics of key genes implicated in B cell activation and TLS formation along the inferred pseudotime trajectory. Density plots confirmed that Naive B cells were enriched at the beginning of the pseudotime trajectory, while Plasma cells were predominantly found at the later stages, with Activated and Memory B cells occupying intermediate positions (**Figure 7F**). Notably, IRF4 expression peaked early in pseudotime, aligning with the Naive B cell state, and declined progressively as cells transitioned toward more differentiated states (Activated, Memory, Plasma) (**Figure 7F-G**). In contrast, key genes essential for TLS maturation and germinal center activity, such as CD83, BCL6, and AICDA, displayed peak expression in the mid-to-late pseudotime trajectory, especially within Activated and Memory B cell populations.

Collectively, these findings suggest a temporally restricted role for IRF4: it is highly expressed during the initial recruitment or activation of immature B cells, potentially promoting TLS initiation, but its downregulation appears essential for subsequent B cell maturation and the establishment of functional TLSs. Persistent IRF4 expression may thus contribute to TLS immaturity, limiting effective anti-tumor immune responses in the ccRCC microenvironment.

### IRF4 Segregating from Mature TLS regions and High Endothelial Venules

**Figure 8:**
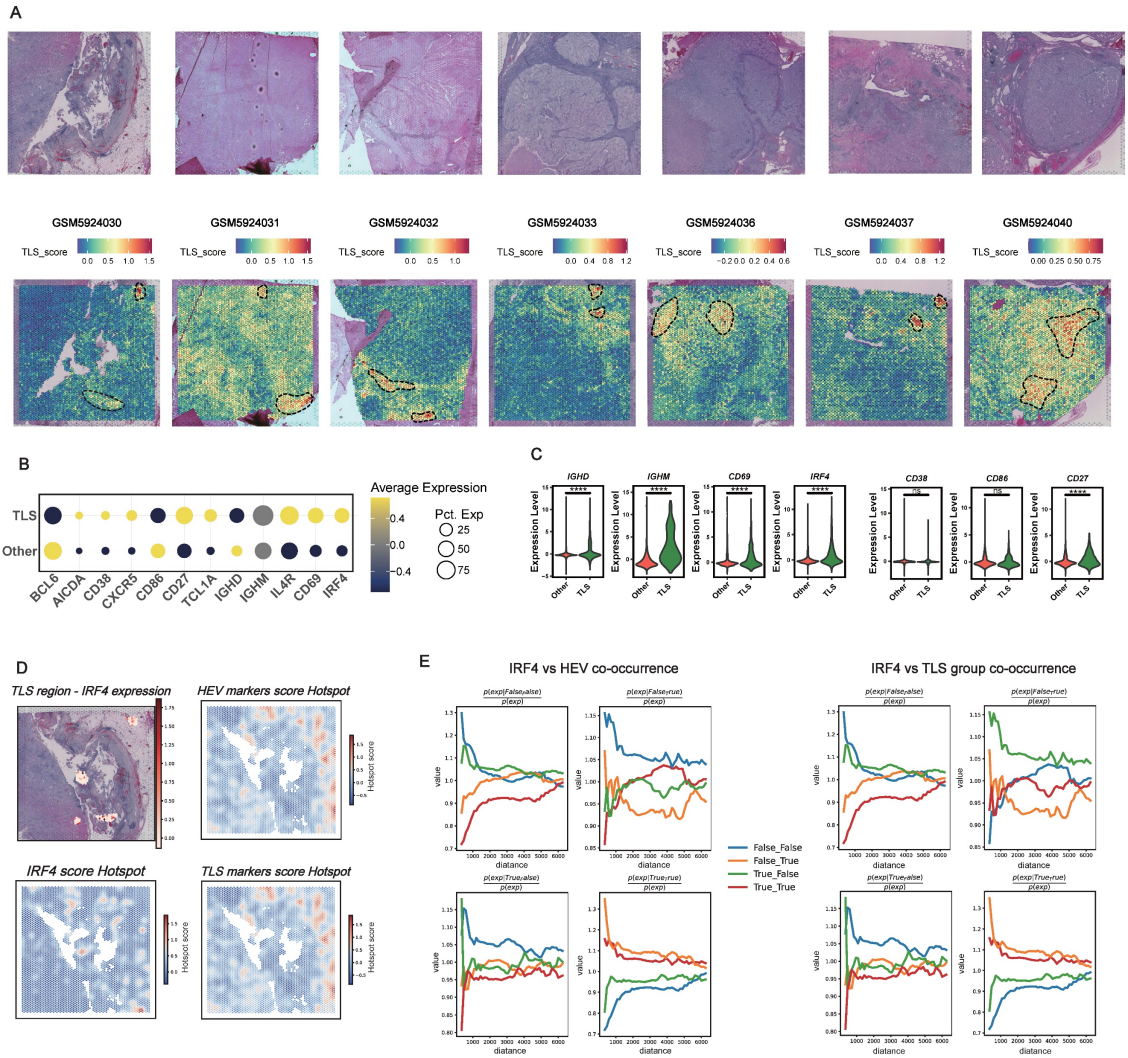
** IRF4 Segregating from Mature TLS regions and High Endothelial Venules.** (A) Identification of TLS regions by TLS score across multiple spatial transcriptomics samples. (B) Dot plot summarizing gene expression patterns in computationally defined TLS versus non-TLS (Other) regions. (C) Violin plots comparing the expression level distribution of selected genes between TLS and non-TLS (Other) regions. (D) Detailed spatial analysis within a representative TLS region, including spatial heatmap of IRF4 expression levels and Hotspot analysis result for IRF4 score, high endothelial venules (HEV) markers score, and TLS markers score. (E) Spatial co-occurrence analysis assessing the spatial relationship between IRF4 expression and HEV or TLS markers score. *, p<0.05; **, p<0.01; ***, p<0.001; ns, not significant.

The single-cell analyses strongly suggested that IRF4 primarily functions during the early phases of B cell recruitment and TLS initiation, rather than supporting their maturation. This raised a critical question: are IRF4-expressing cells spatially localized within immature regions of TLSs, distinct from the functional zones associated with maturation? To address this, we performed spatial transcriptomics analysis 83. Initially, we visualized H&E staining tissue sections alongside spatial heatmaps representing TLS scores (**Figure 8A**). Regions with high TLS scores were computationally identified and demarcated. We then compared the average expression levels of IRF4 and mature TLS-associated genes between the defined TLS regions and adjacent non-TLS regions (“Other”) across all analyzed samples. Dot plot visualizations demonstrated that IRF4, along with most canonical mature TLS markers, exhibited significantly higher average expression within TLS regions compared to non-TLS areas (**Figure 8B**). Statistical validation confirmed the significant upregulation of IRF4 expression within TLS regions (**Figure 8C**).

To investigate the fine-scale spatial organization within TLSs, we conducted spatial hotspot analysis on representative tissue sections (**Figure 8D**). While IRF4 expression was markedly elevated within TLS regions, the spatial distribution of IRF4 hotspots displayed a distinct pattern compared to hotspots derived from high endothelial venule (HEV) markers and TLS maturation gene sets. Specifically, the spatial hotspots for HEV and maturation markers showed considerable overlap, reflecting their co-localization within functional TLS compartments. In contrast, IRF4 expression hotspots were spatially segregated, residing apart from these mature functional zones.

Quantitative spatial co-occurrence analysis further revealed that, despite its overall enrichment within TLS areas, IRF4 lacked strong spatial co-localization with markers of mature TLS zones or HEVs across varying distance scales (**Figure 8E**). This pattern sharply contrasted with the robust co-localization observed among maturation markers themselves, reinforcing the notion that IRF4 is compartmentalized away from functional maturation hubs within the TLS.

These spatial transcriptomics findings aligned closely with our histopathological observations from the FUSCC cohort. IHC analysis confirmed that high IRF4 protein expression was significantly associated with early, immature TLSs (E-TLS). Moreover, tumors characterized by high IRF4 expression and E-TLS dominance exhibited smaller overall TLS areas and markedly reduced CD8⁺ T cell infiltration (**Figure 5B-D**), underscoring the functional relevance of IRF4's spatial and molecular profile in shaping immune cell organization and infiltration.

Taken together, our integrative analysis clarifies the multifaceted role of IRF4 in ccRCC TLS biology. High IRF4 expression, as identified by scRNA-seq, marks immature B cell states and suggests a role in TLS initiation. However, its sustained expression inversely correlates with B cell maturation markers, indicating an inhibitory effect on functional progression. Spatially, IRF4 is enriched within TLSs but remains segregated from mature zones and HEVs, disrupting the spatial architecture necessary for effective TLS maturation. Collectively, these findings suggest that IRF4 fosters TLS immaturity by halting B cell development and impairing spatial organization, thereby contributing to an immunosuppressive tumor microenvironment and weakening anti-tumor immunity.

## Discussion

Previous studies have explored the role of TLSs across various cancer types, shedding light on their relevance to prognosis and treatment responses 84-86. However, research specifically addressing the relationship between TLSs and renal cell carcinoma, particularly ccRCC, remains limited. Our study aimed to address this gap by investigating the complex interplay between TLS-related genes and the pathophysiology of ccRCC. Through an extensive integrative analysis, we examined genetic alterations, their associations with patient survival, and their influence on the TME, offering an in-depth understanding of the immunological landscape in ccRCC.

Building on this framework, our comprehensive profiling of TLS-related genes in ccRCC provided key insights into their expression patterns, genetic variations, and clinical significance, laying the groundwork for improved prognostication and potential therapeutic interventions. Specifically, we analyzed the expression profiles, copy number variation (CNV) frequencies, somatic mutation rates, and interconnections of 39 TLS-related genes. Using consensus clustering, we stratified patients into three TLSClusters (A-C), each showing distinct survival outcomes, TME characteristics, enriched KEGG pathways, immune cell infiltration profiles, and immune checkpoint (ICP) expression levels. From these clusters, we identified 549 differentially expressed genes (DEGs), of which 155 were determined to have prognostic value through univariate Cox regression analysis. These genes further enabled the classification of patients into three GeneClusters (A-C), which also demonstrated significant differences in both clinical and immunological features. Collectively, these findings underscore the critical role of TLSs in shaping the immune architecture of ccRCC and influencing patient outcomes.

To enhance clinical applicability, we developed a prognostic signature, termed the RiskScore, derived from the 155 prognostic genes using LASSO and multivariate Cox regression analyses. This five-gene signature (CCL22, LIPA, LOXL1, ADAM8, and SAA1) showed strong correlations with patient survival. Notably, CCL22, a chemokine enriched in TLSs 87, has been implicated in promoting regulatory T cell (Treg) recruitment and suppressive activity in hepatocellular carcinoma 88. LOXL1 contributes to extracellular matrix remodeling and TME disruption 89, while ADAM8 facilitates interactions between tumor cells and macrophages 90, playing a prominent role in several malignancies 91. SAA1 promotes leukocyte recruitment, either directly or via chemotactic cascades 92, and has been linked to ccRCC progression by modulating mast cells and PD-L1 expression 93. To further support clinical utility, we integrated the RiskScore with key clinical variables into a nomogram, which demonstrated strong predictive accuracy and calibration in survival prediction.

Our findings align with, and extend, the growing body of literature emphasizing the importance of TLSs in modulating tumor progression and shaping immunotherapeutic responses. For example, Ling et al. employed a deep-learning TLS classifier on H&E-stained slides, demonstrating that mature TLSs serve as independent prognostic markers in esophageal squamous cell carcinoma 94. Ruffin et al. showed that the spatial organization of immune cells indicative of TLSs with germinal centers correlated with better prognosis in HPV-positive head and neck squamous cell carcinoma 85. Additionally, Kinker et al. find that the gene signature indicative of mature TLSs is more prevalent in pre-treatment biopsy samples from pancreatic ductal adenocarcinoma patients with extended survival following various chemoimmunotherapy treatments 95. Within renal cell carcinoma, TLSs have drawn growing attention. Mylan et al. reported that the presence of TLSs was associated with enhanced immunotherapy responses, potentially by sustaining B cell maturation 83, while Dai et al. identified intratumoral CXCL13^+^CD8^+^ T cells, a TLS component, as markers of poor prognosis in ccRCC^96^. Moreover, Xu et al. further demonstrated that ccRCC patients undergoing anti-PD-1/PD-L1 therapy exhibited improved survival and response rates when intratumoral TLSs and SFL-TLSs were present 73. Our TLS-based prognostic signature thus builds on these findings, offering a refined tool to distinguish between favorable and unfavorable clinical trajectories.

ccRCC patients with SFL-TLS have a better prognosis compared to those with no SFL-TLS, indicating the maturity of TLSs matters when assessing the patient's condition 39. Therefore, we investigated the characteristics of TLS maturation heterogeneity using mIF. While the overall TLS landscape and the derived signature provide valuable prognostic information, understanding the roles of specific regulatory genes within this context is crucial for deeper mechanistic insight. While the overall TLS signature provided prognostic insights, we focused on the mechanistic role of key regulatory genes, particularly IRF4, a central transcription factor in B cell biology. Consistent with prior reports in other cancers such as OSCC 51, IRF4 expression was higher in tumor tissues compared to normal tissues. We observed that IRF4 was predominantly enriched in E-TLS compared to PFL-TLS and SFL-TLS, with higher IRF4 levels correlating with smaller TLS areas, reduced CD8^+^ T cell infiltration, and worse overall and progression-free survival. IRF4 is known to be a critical regulator of lymphocyte development and function, and typically acts as a master regulator in B cells 97. Its precise role can be context-dependent, influencing differentiation, activation, and potentially exhaustion 45. Functionally, IRF4 knockdown in ccRCC cells resulted in diminished proliferation, invasion, and migration alongside increased apoptosis, suggesting that IRF4 exerts a pro-tumorigenic effect and impedes the development of a mature, anti-tumor immune microenvironment.

Mechanistically, we found that IRF4 knockdown was associated with reduced expression of CXCL13 and BCL6, alongside increased PD-L1 expression. CXCL13, a key chemokine for TLS formation, orchestrates the recruitment of B and T cells to the TME 98. CXCL13 is also reported to serve as a positive prognostic indicator in ovarian cancer 99. However, in ccRCC, CXCL13-CXCR5 interactions paradoxically promote tumor proliferation and migration 100, aligning with our observation that reducing IRF4 lowers pro-tumorigenic CXCL13. Elevating BCL6 levels is crucial for initiating the GC reaction, given that BCL6-deficient GC precursor B cells cannot migrate into the follicle 101. In diffuse large B cell lymphoma, BCL6 suppresses cell death genes, hence promoting tumor growth, while this progression can be eliminated by inhibiting BCL6^102^. Interestingly, IRF4 can both stimulate and inhibit BCL6 transcription depending on context 103,104. Our results suggested that IRF4 promotes BCL6 and thereby may contribute to tumor progression in ccRCC. In advanced ccRCC, PD-L1 expression is prevalent at both primary and metastatic locations 105. When PD-L1 engages PD1, it inhibits the PI3K-AKT and RAS-MEK-ERK pathways, suppressing T-cell expansion and tumor-killing effects 106. Our investigation indicated IRF4 may enhance this process in ccRCC, as higher PD-L1 levels were observed in the absence of IRF4.

To elucidate how high IRF4 expression links to immature TLS phenotypes, we leveraged single-cell RNA sequencing to map B cell subpopulations within the ccRCC TME. Pseudotime trajectory analysis indicated a progression from naive to plasma-like B cells, with IRF4 expression peaking at early stages and declining along the maturation path. In contrast, markers of TLS maturation and germinal center activity were most prominent in later stages. This inverse relationship suggests that while IRF4 promotes early B cell presence and TLS initiation, its sustained expression may hinder functional maturation, preventing the formation of germinal center-like structures essential for effective anti-tumor immunity. These findings are consistent with the known stage-specific functions of IRF4 in B cell differentiation 97,107, and participates in networks orchestrating the antibody response 108. Our data suggest that in ccRCC, sustained high IRF4 may lock B cells in an early or alternative state, preventing full maturation within TLSs.

Complementing the single-cell perspective, our spatial transcriptomics analysis provided crucial context regarding IRF4's location within the tumor architecture. We confirmed that IRF4 expression is significantly upregulated within computationally defined TLS regions compared to adjacent non-TLS areas. However, a finer-scale spatial analysis using hotspot identification and co-occurrence modeling revealed a distinct pattern. While IRF4 expression was elevated within the TLS, its spatial hotspots showed significant segregation from hotspots associated with markers of HEVs and TLS maturation gene sets. This indicates that the cells expressing high levels of IRF4, while part of the TLS aggregate, are often located in regions spatially distinct from the mature, functional compartments characterized by active immune cell recruitment and germinal center-like activity. This spatial segregation reinforces the hypothesis derived from scRNA-seq and IHC data: high IRF4 expression in ccRCC TLSs is associated with structures or cellular states that are distinct from, and potentially inhibitory to, the development of fully mature, SFL-TLS like structures. These observations resonate with prior studies highlighting IRF4's role in B cell positioning and function within lymphoid tissues 109.

Despite these important insights, our study has several limitations. As a retrospective analysis, it carries inherent risks of selection and information biases. Additionally, our reliance on publicly available datasets may limit generalizability across diverse patient populations. Importantly, while our findings highlight correlations between TLS-related gene expression, IRF4 levels, and clinical outcomes, they remain largely associative. Future studies, including prospective cohorts and mechanistic experiments, are warranted to validate these associations and further elucidate the causal roles of TLSs and IRF4 in ccRCC progression.

## Conclusion

In conclusion, this study established a TLS-related signature in ccRCC, providing insights into TME features and tumor progression. We establish a robust TLS-based gene signature predictive of patient outcomes and identify IRF4 as a key regulator that impairs TLS maturation by arresting B cell development and disrupting spatial organization. Together, these findings highlight both the TLS signature and IRF4 as promising prognostic markers and therapeutic targets, offering new avenues to refine immunotherapy strategies in ccRCC.

## Supplementary Material

Supplementary figures.

Supplementary tables.

## Abbreviations

AUCs: area under the curves
ccRCC: clear cell renal cell carcinoma
CI: confidence interval
CNV: copy number variation
CPTAC: Clinical Proteomic Tumor Analysis Consortium
CR/PR: complete response/partial response
DAPI: 4',6-diamidino-2-phenylindole
DCA: decision curve analysis
DC: dendritic cell
DEG: differential expression gene
DFS: disease-free survival
ECL: enhanced chemiluminescence
EMBL: European Molecular Biology Laboratory
E-TLS: early tertiary lymphoid structures
FC: fold change
FDC: follicular DC
FPKM: per million mapped reads
FUSCC: Fudan University Shanghai Cancer Center
GC: germinal center
GEO: Gene Expression Omnibus
GO: Gene Ontology
GSVA: gene set variation analysis
H&E: Hematoxylin and eosin
HCC: hepatocellular carcinoma
HEV: high endothelial venule
HRP: horseradish peroxidase
ICGC: International Cancer Genome Consortium
ICI: immune checkpoint inhibitor
ICPs: immune checkpoints
IHC: immunohistochemistry
IPS: Immunophenotype Score
IRF4: interferon regulatory factor 4
KEGG: Kyoto Encyclopedia of Genes and Genomes
K-M: Kaplan-Meier
LASSO: least absolute shrinkage and selector operation
mIF: Multiplex Immunofluorescence
MM: multiple myeloma
NK: Natural Killer
OS: overall survival
OSCC: oral squamous cell carcinoma
PBS: Phosphate-Buffered Saline
PCA: Principal component analysis
PD-1: Programmed cell death protein 1
PD-L1: Programmed death-ligand 1
PFL-TLS: primary follicle-like tertiary lymphoid structures
PFS: progression-free survival
PPI: protein-protein interaction
RCC: renal cell carcinoma
ROC: receiver operating characteristic
scRNA-seq: Single-cell RNA sequencing
SD/PD: stable disease/progressive disease
SDS-PAGE: Sodium dodecyl-sulfate polyacrylamide gel electrophoresis
SFL-TLS: secondary follicle-like tertiary lymphoid structures
siRNA: small interfering RNA
SLO: secondary lymphoid organ
ssGSEA: single-sample gene set enrichment analysis
ST: spatial transcriptomic
TBST: Tris-Buffered Saline with Tween 20
TCGA: the Cancer Genome Atlas
TCIA: The Cancer Immunome Atlas
Teff: effector T
TFH: T follicular helper
TH1: T helper 1
TIDE: tumor immune dysfunction and exclusion
TILs: tumor-infiltrating lymphocytes
TLS: tertiary lymphoid structures
TMB: tumor mutation burden
TME: tumor microenvironment
TPM: transcripts per million
Treg: regulatory T cell
t-SNE: t-Distributed Stochastic Neighbor Embedding
uniCox: univariate Cox regression
