# Supplementary material for: Interferon Regulatory Factor 4 Recruits Immature B Cells to Signal Tertiary Lymphoid Structure Immaturity and Progression of Clear Cell Renal Cell Carcinoma
Source: Int J Biol Sci. 2025 Jun 9;21(9):3827–51. doi: 10.7150/ijbs.113737 (PMC12210385; doi:10.7150/ijbs.113737)
Supplement: Supplementary file 1 — Supplementary figures. [file ijbsv21p3827s1.pdf]

SUPPLEMENTARY FIGURE 1. K-M curves of the ccRCC patient subgroups stratified by the expression levels of TLS genes.

(A-Z) K-M analyses for OS of ccRCC patient subgroups based on the RNA expressions of CCL5, CCL8, CCL18, CCL19, CCL20, CCL21, CCR5, CD4, CD5, CD38, CD40, CD200, CSF2, CXCL9, CXCL10, CXCL11, CXCL13, IGSF6, IL1R2, IL2RA, IRF4, MS4A1, SGPP2, SH2D1A, TNFRSF17 and TRAF6.

SUPPLEMENTARY FIGURE 2. Identification of TLS patterns based on the expression levels of the 39 TLS genes.

(A) Copy number variations (CNVs) frequencies of the 39 TLS genes.
(B) Frequency of somatic mutations and classification of the 39 TLS genes.
(C) Distribution of CNVs of 39 TLS genes across 23 chromosomes.
(D-K) Consensus matrices of ccRCC patients from k = 2 to k = 9.
(L-N) The cumulative distribution function (CDF) curves plot, delta plot, and tracking plot corresponding to the consensus matrices from k = 2 to k = 9.

SUPPLEMENTARY FIGURE 3. Clinicopathological and biological characteristics of three subgroups defined by the 39 TLS genes in ccRCC.

(A) Principal component analysis (PCA) showing unique dispersion among TLSClusters.
(B) Differences in stromal, immune, and ESTIMATE scores among TLSClusters.
(C) Heatmap of 39 TLS gene expressions and clinical characteristics among three distinct TLSClusters.
(D-F) Gene set variation analysis (GSVA) of activated biological pathways among TLSClusters.
(G) Immune cell infiltration differences among TLSClusters.
(H) Expressions of ICPs in TLSClusters.
(I-L) The distribution of clinical characteristics (age, gender, grade, and stage) among ccRCC patients in TLSClusters.
*, $p<0.05$; **, $p<0.01$; ***, $p<0.001$; ns, not significant.

SUPPLEMENTARY FIGURE 4. Identification of GeneClusters based on the 155 prognosis-related genes.

(A) The protein-protein interaction (PPI) network illustrating the interactions among the top 50 DEGs within the three TLSClusters.
(B-C) Functional enrichment analyses of Kyoto encyclopedia of genes and genomes (KEGG) and gene ontology (GO) analyses of DEGs.
(D-K) Consensus matrices of ccRCC patients from k = 2 to k = 9.
(L-N) The cumulative distribution function curves plot, delta plot, and tracking plot corresponding to the consensus matrices from k = 2 to k = 9.

SUPPLEMENTARY FIGURE 5. Clinicopathological and biological characteristics of GeneClusters.

(A) Heatmap showing variations in clinical features among GeneClusters.

(B) Differences in the TME scores among GeneClusters.
(C-E) Heatmaps showing varied activation statuses of biological pathways within GeneClusters by GSVA.
(F) The abundance of infiltrating immune cell types within GeneClusters.
(G) Expressions of ICPs in GeneClusters.
(H-K) The distribution of clinical characteristics (age, gender, grade, and stage) among ccRCC patients in GeneClusters.
*, $p<0.05$; **, $p<0.01$; ***, $p<0.001$; ns, not significant.

SUPPLEMENTARY FIGURE 6. TLS-Associated Predictive Signature Development and its correlation between clinical characteristics.
(A) The ranked dots and scatter plot illustrating the RiskScore distribution and survival status, accompanied by a heatmap depicting the expression profiles of the five chosen genes.
(B) The receiver operating characteristic (ROC) curves demonstrating the sensitivity and specificity of 1-, 3-, and 5-year OS based on the RiskScore.
(C) The alluvial diagram illustrating the distribution of patients among different clusters, RiskGroups, and clinical outcomes.
(D) Disparities in patients' RiskScore among the three TLSClusters.
(E) Variations in patients' RiskScore among the three GeneClusters.
(F-G, J-K, and N-O) K-M analyses for OS of patients in high and low RiskGroups stratified by T stage, TNM stage, and tumor grade.
(H-I, L-M, and P-Q) The distribution of clinical characteristics (T stage, TNM stage, and tumor grade) among ccRCC patients in high and low RiskGroups.

SUPPLEMENTARY FIGURE 7. Establishment of a Prognostic Prediction Nomogram.
(A) TLS-associated nomogram predicting the 1-, 3-, and 5-year OS of ccRCC patients.
(B) Calibration curves for validation of the nomogram.
(C-E) ROC curves for 1-, 3-, and 5-year OS prediction of the nomogram.
(F-H) Decision curve analysis (DCA) for 1-, 3-, and 5-year OS prediction of the nomogram.

Figure S1

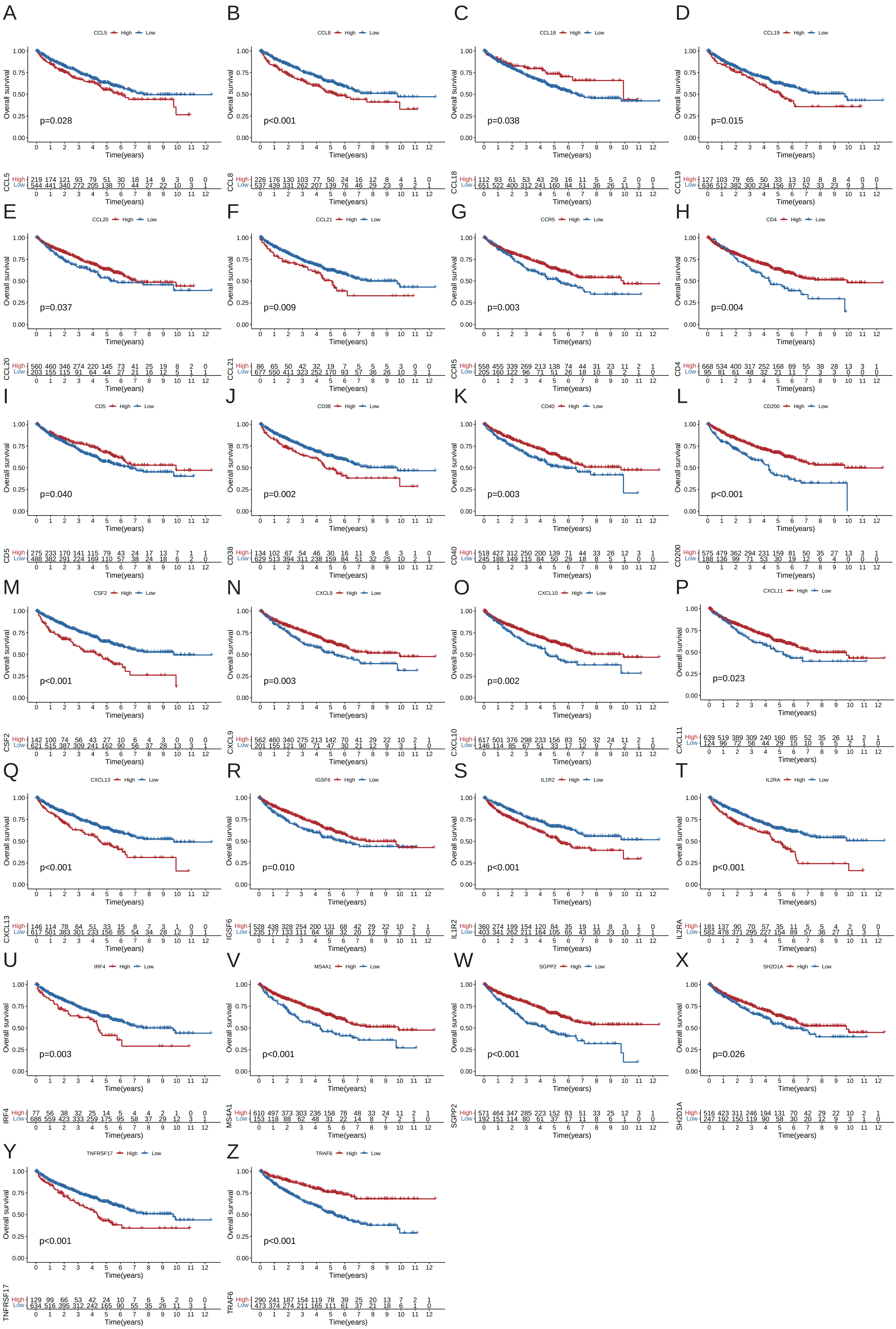
A
CCL5 High Low
p=0.028
B
CCL8 High Low
p<0.001
C
CCL18 High Low
p=0.038
D
CCL19 High Low
p=0.015
E
CCL20 High Low
p=0.037
F
CCL21 High Low
p=0.009
G
CCR5 High Low
p=0.003
H
CD4 High Low
p=0.004
I
CD5 High Low
p=0.040
J
CD38 High Low
p=0.002
K
CD40 High Low
p=0.003
L
CD200 High Low
p<0.001
M
CSF2 High Low
p<0.001
N
CXCL9 High Low
p=0.003
O
CXCL10 High Low
p=0.002
P
CXCL11 High Low
p=0.023
Q
CXCL13 High Low
p<0.001
R
IGSF6 High Low
p=0.010
S
IL1R2 High Low
p<0.001
T
IL2RA High Low
p<0.001
U
IRF4 High Low
p=0.003
V
MS4A1 High Low
p<0.001
W
SGPP2 High Low
p<0.001
X
SH2D1A High Low
p=0.026
Y
TNFRSF17 High Low
p<0.001
Z
TRAF6 High Low
p<0.001
Overall survival
Time(years)

Figure S2

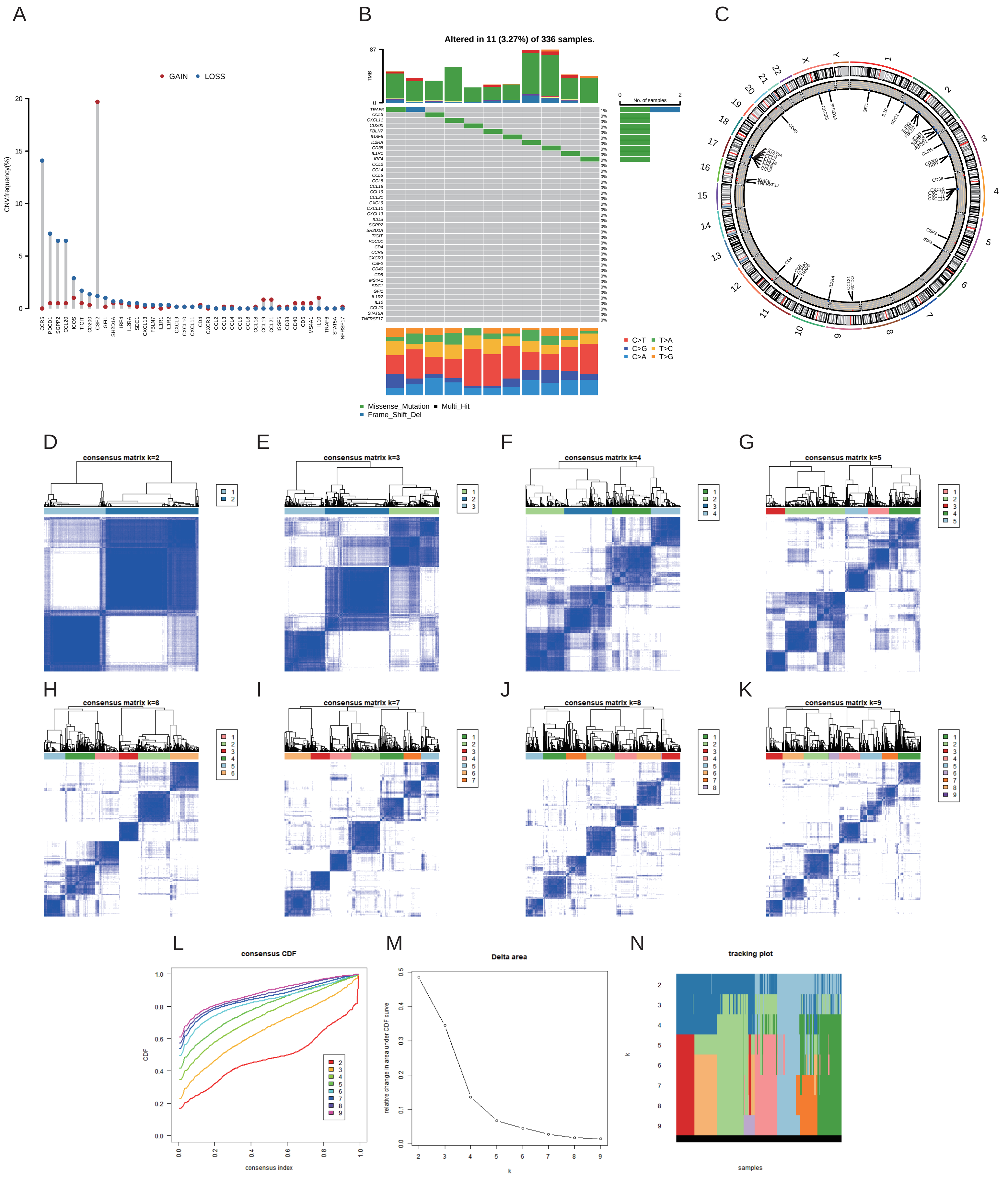
A
B
C
GAIN
LOSS
CNV.frequency(%)
Altered in 11 (3.27%) of 336 samples.
TMB
No. of samples
C>T
T>A
C>G
T>C
C>A
T>G
Missense_Mutation
Multi_Hit
Frame_Shift_Del
D
E
F
G
H
I
J
K
consensus matrix k=2
consensus matrix k=3
consensus matrix k=4
consensus matrix k=5
consensus matrix k=6
consensus matrix k=7
consensus matrix k=8
consensus matrix k=9
L
M
N
consensus CDF
CDF
consensus index
Delta area
relative change in area under CDF curve
k
tracking plot
samples

Figure S3

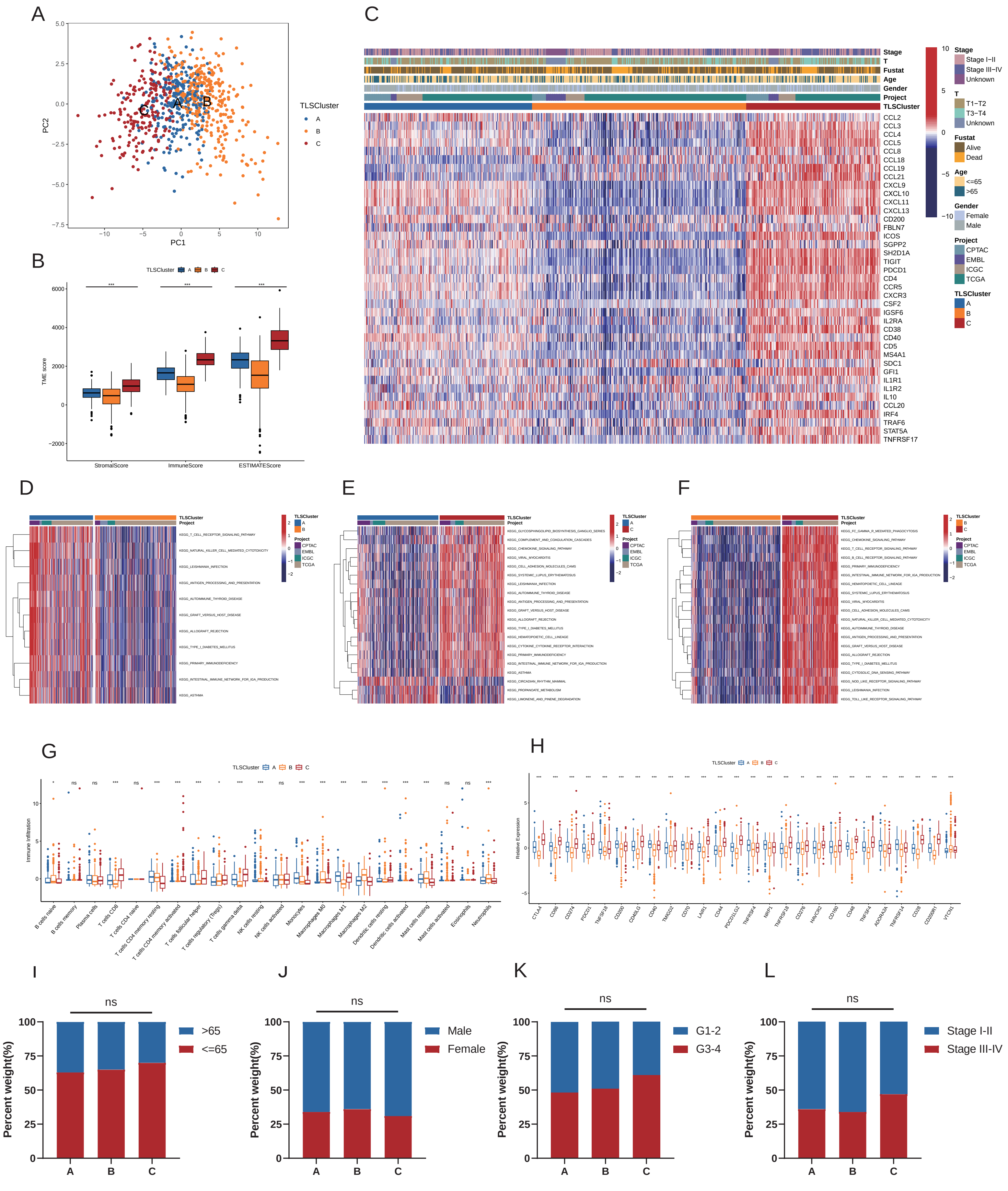

A
B
C
D
E
F
G
H
I
J
K
L
PC1
PC2
TLSCluster
TME score
StromalScore
ImmuneScore
ESTIMATEScore
Stage
T
Fustat
Age
Gender
Project
Stage I−II
Stage III−IV
Unknown
T1−T2
T3−T4
Alive
Dead
<=65
>65
Female
Male
CPTAC
EMBL
ICGC
TCGA
Immune Infiltration
Relative Expression
Percent weight(%)
ns
G1-2
G3-4
Stage I-II
Stage III-IV

Figure S4

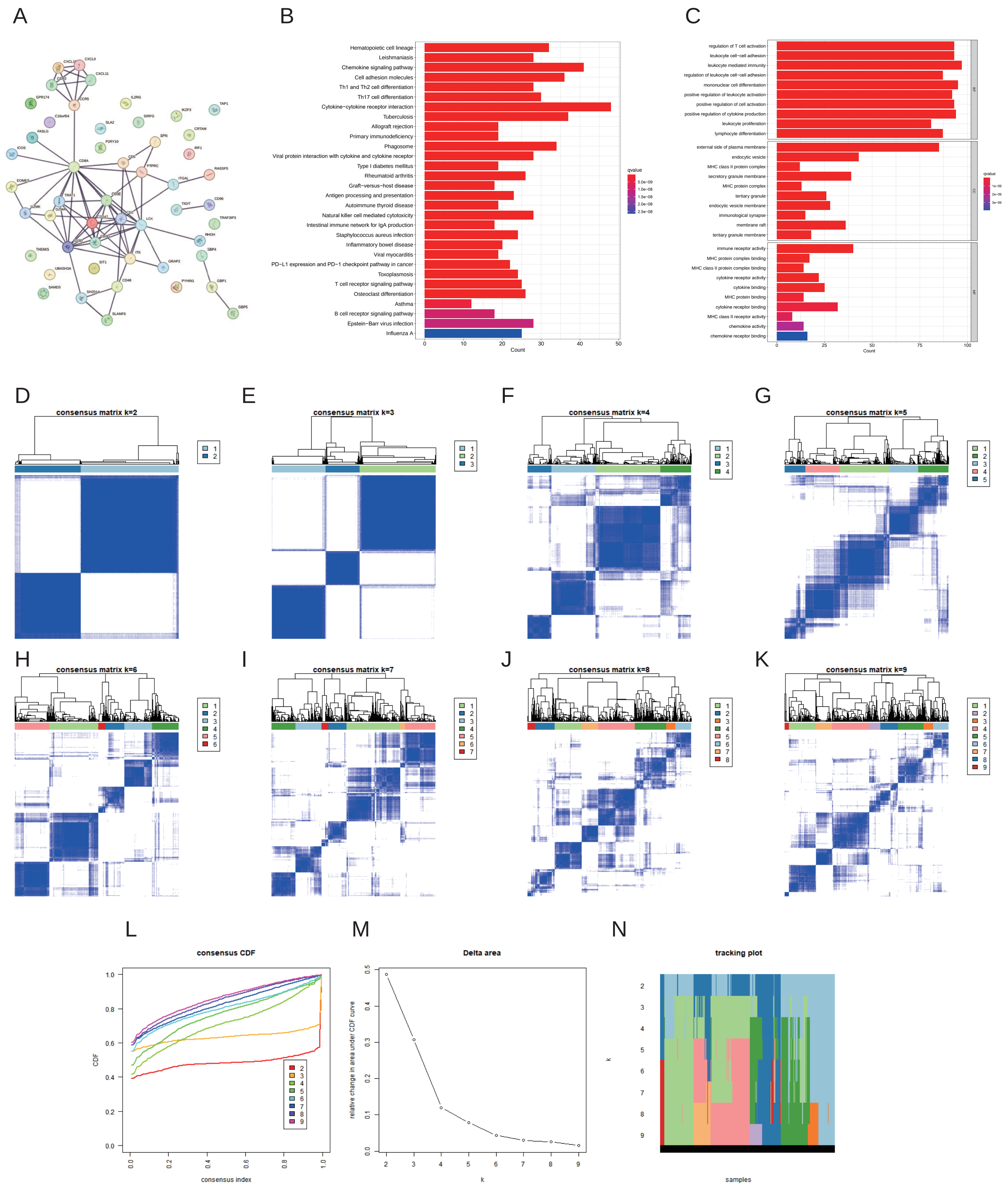
A
B
C
Hematopoietic cell lineage
Leishmaniasis
Chemokine signaling pathway
Cell adhesion molecules
Th1 and Th2 cell differentiation
Th17 cell differentiation
Cytokine−cytokine receptor interaction
Tuberculosis
Allograft rejection
Primary immunodeficiency
Phagosome
Viral protein interaction with cytokine and cytokine receptor
Type I diabetes mellitus
Rheumatoid arthritis
Graft−versus−host disease
Antigen processing and presentation
Autoimmune thyroid disease
Natural killer cell mediated cytotoxicity
Intestinal immune network for IgA production
Staphylococcus aureus infection
Inflammatory bowel disease
Viral myocarditis
PD−L1 expression and PD−1 checkpoint pathway in cancer
Toxoplasmosis
T cell receptor signaling pathway
Osteoclast differentiation
Asthma
B cell receptor signaling pathway
Epstein−Barr virus infection
Influenza A
Count
qvalue
regulation of T cell activation
leukocyte cell−cell adhesion
leukocyte mediated immunity
regulation of leukocyte cell−cell adhesion
mononuclear cell differentiation
positive regulation of leukocyte activation
positive regulation of cell activation
positive regulation of cytokine production
leukocyte proliferation
lymphocyte differentiation
external side of plasma membrane
endocytic vesicle
MHC class II protein complex
secretory granule membrane
MHC protein complex
tertiary granule
endocytic vesicle membrane
immunological synapse
membrane raft
tertiary granule membrane
immune receptor activity
MHC protein complex binding
MHC class II protein complex binding
cytokine receptor activity
cytokine binding
MHC protein binding
cytokine receptor binding
MHC class II receptor activity
chemokine activity
chemokine receptor binding
D
E
F
G
consensus matrix k=2
consensus matrix k=3
consensus matrix k=4
consensus matrix k=5
H
I
J
K
consensus matrix k=6
consensus matrix k=7
consensus matrix k=8
consensus matrix k=9
L
M
N
consensus CDF
CDF
consensus index
Delta area
relative change in area under CDF curve
k
tracking plot
samples

Figure S5

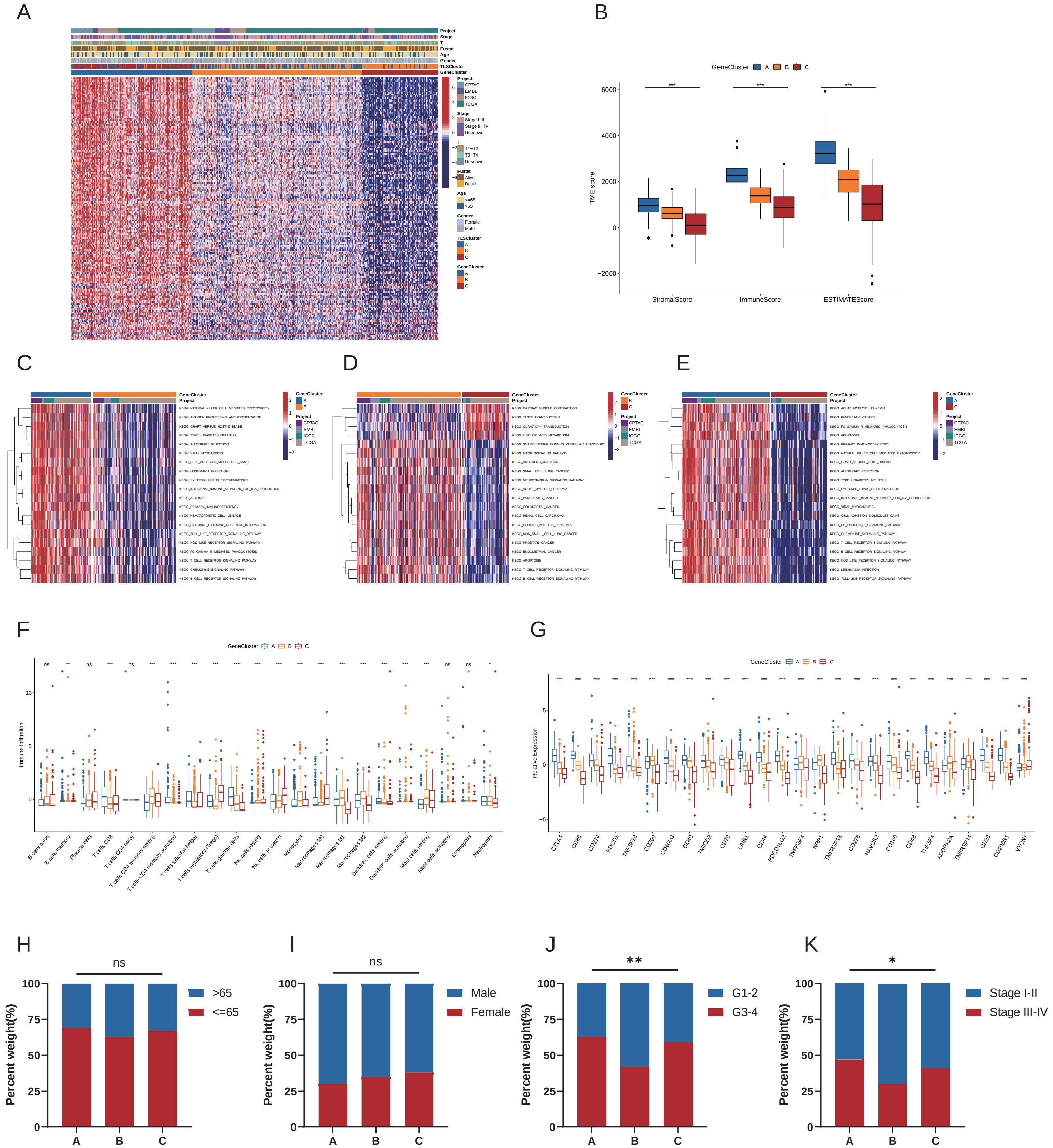

A
B
C
D
E
F
G
H
I
J
K
GeneCluster A B C
TME score
StromalScore
ImmuneScore
ESTIMATEScore
Immune Infiltration
Relative Expression
Percent weight(%)
>65
<=65
Male
Female
G1-2
G3-4
Stage I-II
Stage III-IV
ns
**
*

Figure S6

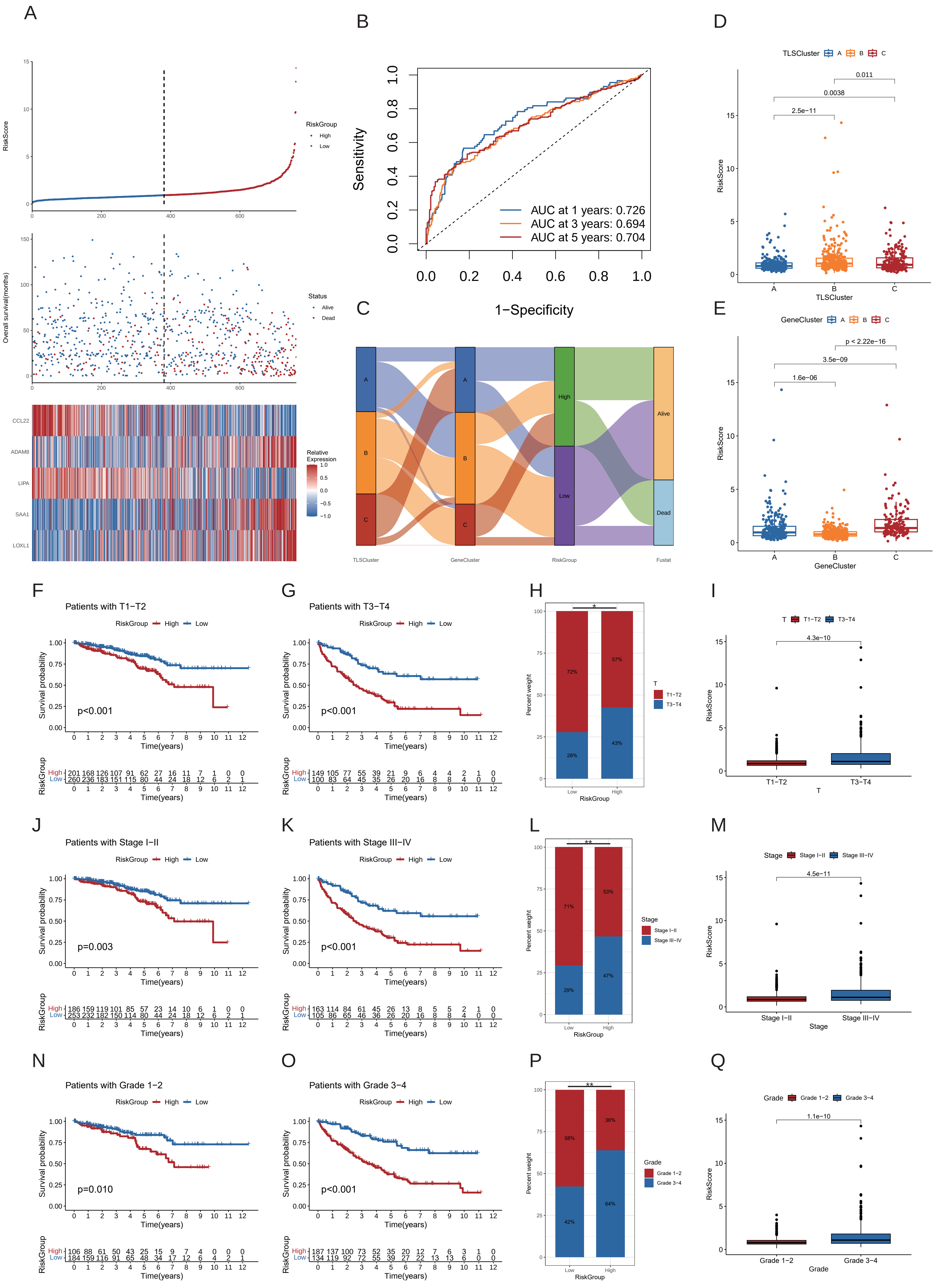
A
B
D
C
E
F
G
H
I
J
K
L
M
N
O
P
Q
RiskScore
RiskGroup
High
Low
Overall survival(months)
Status
Alive
Dead
CCL22
ADAM8
LIPA
SAA1
LOXL1
Relative Expression
Sensitivity
1−Specificity
AUC at 1 years: 0.726
AUC at 3 years: 0.694
AUC at 5 years: 0.704
TLSCluster
GeneCluster
Fustat
2.5e−11
0.0038
0.011
1.6e−06
3.5e−09
p < 2.22e−16
Patients with T1−T2
Patients with T3−T4
Patients with Stage I−II
Patients with Stage III−IV
Patients with Grade 1−2
Patients with Grade 3−4
Survival probability
Time(years)
p<0.001
p=0.003
p=0.010
Percent weight
T1−T2
T3−T4
Stage I−II
Stage III−IV
Grade 1−2
Grade 3−4
4.3e−10
4.5e−11
1.1e−10

Figure S7

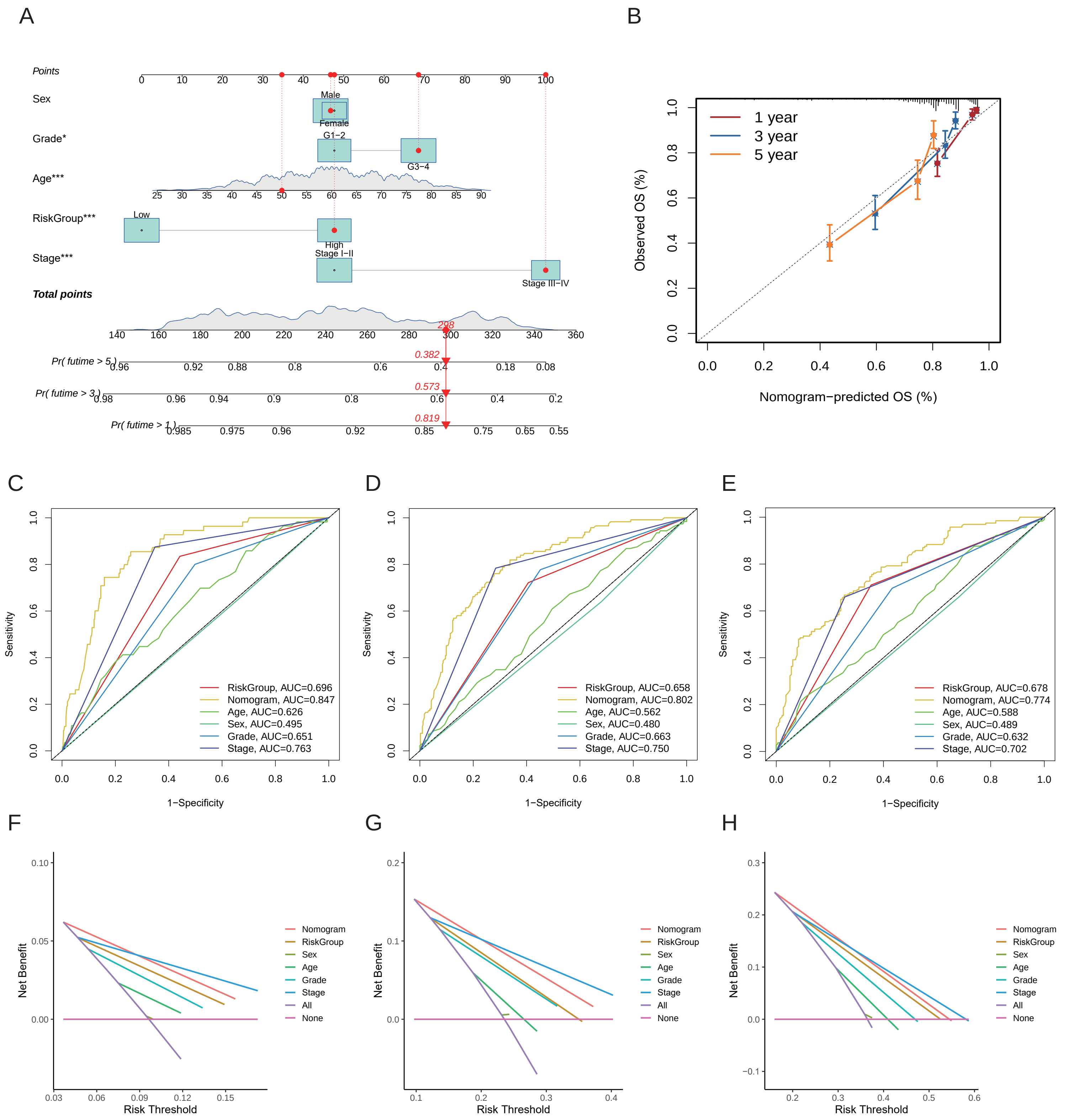
A
Points
Sex
Male
Female
Grade*
G1−2
G3−4
Age***
RiskGroup***
Low
High
Stage***
Stage I−II
Stage III−IV
Total points
298
Pr(futime > 5)
0.382
Pr(futime > 3)
0.573
Pr(futime > 1)
0.819
B
1 year
3 year
5 year
Observed OS (%)
Nomogram−predicted OS (%)
C
RiskGroup, AUC=0.696
Nomogram, AUC=0.847
Age, AUC=0.626
Sex, AUC=0.495
Grade, AUC=0.651
Stage, AUC=0.763
Sensitivity
1−Specificity
D
RiskGroup, AUC=0.658
Nomogram, AUC=0.802
Age, AUC=0.562
Sex, AUC=0.480
Grade, AUC=0.663
Stage, AUC=0.750
Sensitivity
1−Specificity
E
RiskGroup, AUC=0.678
Nomogram, AUC=0.774
Age, AUC=0.588
Sex, AUC=0.489
Grade, AUC=0.632
Stage, AUC=0.702
Sensitivity
1−Specificity
F
G
H
Nomogram
RiskGroup
Sex
Age
Grade
Stage
All
None
Net Benefit
Risk Threshold
